# Neutrophil-like pH-responsive pro-efferocytic nanoparticles improve neurological recovery by promoting erythrophagocytosis after intracerebral hemorrhage

**DOI:** 10.7150/thno.90370

**Published:** 2024-01-01

**Authors:** Linfeng Fan, Lulu Jin, Tianchi Tang, Yonghe Zheng, Zihang Chen, Haopu Lin, Chao Ding, Tingting Wang, Huaijun Chen, Yinghan Guo, Chaoran Xu, Hang Zhou, Xinyan Wu, Xiongjie Fu, Feng Yan, Zhengwei Mao, Gao Chen

**Affiliations:** 1 Department of Neurosurgery, The Second Affiliated Hospital of Zhejiang University School of Medicine, Zhejiang University, Hangzhou, 310016, China.; 2 Key Laboratory of Precise Treatment and Clinical Translational Research of Neurological Diseases, Hangzhou, 310016, China.; 3 MOE Key Laboratory of Macromolecular Synthesis and Functionalization, Department of Polymer Science and Engineering, Zhejiang University, Hangzhou, 310027, China.

**Keywords:** intracranial hemorrhage, erythrophagocytosis, pH-responsive, neutrophil-like nanoparticles, neurological recovery

## Abstract

**Rationale:** Intracerebral hemorrhage (ICH) is a devastating cerebrovascular disease resulting from blood extravasating into the brain parenchyma. Escalation of erythrophagocytosis (a form of efferocytosis), avoiding the consequent release of the detrimental erythrocyte lysates, may be a promising target of ICH management. The ADAM17 inhibitor and liver X receptor (LXR) agonist could promote efficient efferocytosis and injury repair. Nevertheless, the poor bioavailability and restriction of the blood-brain barrier (BBB) hinder their application. Therefore, it is needed that biocompatible and smart nanoplatforms were designed and synthesized to realize effective therapy targeting erythrophagocytosis.

**Methods:** We first assessed the synergistic effect of therapeutic GW280264X (an ADAM17 inhibitor) and desmosterol (an LXR agonist) on erythrophagocytosis* in vitro*. Then a pH-responsive neutrophil membrane-based nanoplatform (NPEOz) served as a carrier to accurately deliver therapeutic GW280264X and desmosterol to the damaged brain was prepared via co-extrusion. Afterwards, their pH-responsive performance was valued *in vitro* and targeting ability was assessed through fluorescence image *in vivo.* Finally, the pro-erythrophagocytic and anti-neuroinflammatory ability of the nanomedicine and related mechanisms were investigated.

**Results:** After the synergistical effect of the above two drugs on erythrophagocytosis was confirmed, we successfully developed neutrophil-disguised pH-responsive nanoparticles to efficiently co-deliver them. The nanoparticles could responsively release therapeutic agents under acidic environments, and elicit favorable biocompatibility and ability of targeting injury sites. D&G@NPEOz nanoparticles enhanced erythrophagocytosis through inhibiting shedding of the efferocytotic receptors MERTK/AXL mediated by ADAM17 and accelerating ABCA-1/ABCG-1-mediated cholesterol efflux regulated by LXR respectively. In addition, the nano-formulation was able to modulate the inflammatory microenvironment by transforming efferocytes towards a therapeutic phenotype with reducing the release of proinflammatory cytokines while increasing the secretion of anti-inflammatory factors, and improve neurological function.

**Conclusions:** This biomimetic nanomedicine is envisaged to offer an encouraging strategy to effectively promote hematoma and inflammation resolution, consequently alleviate ICH progression.

## Introduction

Intracerebral hemorrhage (ICH) is one of the most devastating subtypes of cerebrovascular diseases, which threatens millions of people in the world annually, with a high morbidity and mortality, yet without an effective therapy 1-4. The mass effect that rapidly formed hematoma mechanically deforms and squeezes the brain tissue, causes the primary injury. Subsequently erythrocytes lysis releases large amounts of detrimental products such as free iron and heme to induce the secondary injury which is characterized by neuroinflammation, oxidative stress and so on 5, 6. Increasing results have revealed that early clearance of the hematoma may be a promising target of ICH management 5, 7, 8. Surgery is an important way to remove hematoma. However, this approach involves invasive craniotomy and intracranial catheterization, with high risks of infection and additional tissue damage 9. Additionally, there are numerous patients, with serious diabetes or hypertension and less bleeding, who are not suitable for surgery 10. Therefore, a workable pharmacological strategy to accelerate the endogenous removal of hematoma is exigently demanded.

Microglia/macrophage, as professional phagocytes in the central nervous system (CNS), are activated and recruited rapidly to insult sites for efferocytosis by which apoptotic cells are cleared 11, 12. It has been demonstrated that accelerating erythrophagocytosis by microglia/macrophage prior to erythrocyte lysis and reducing the subsequent introduction of toxic hemolytic products could protect against brain insults, modulate the inflammatory environment and improve neurologic deficits in murine ICH 2, 7, 13. In such context, erythrophagocytosis was carefully mediated by a set of surface receptors that discerned externalized phosphatidylserine expressed on apoptotic erythrocytes 14-16. AXL/MERTK were the key phosphatidylserine receptors. Unfortunately, they were prone to be cleaved by a disintegrin and metalloproteinases such as ADAM17 17-20. In turn, genetic or pharmacological inhibition of ADAM17 promoted efficient efferocytosis and injury repair 18, 21-23. Sommer *et al.* suggested pharmacological inhibition of ADAM17 promoted microglia phagocytosis and neurological functional recovery after spinal cord injury 24. However, excessive accumulation of phagocytic cargoes such as cholesterol, which were not digested in time after efferocytosis, adversely affected the process in turn, and even resulted in abnormal foam cells formation 11, 25, 26. Fortunately, phagocytes could sense massive cholesterol and then mediate their efflux by the nuclear receptors liver X receptor (LXR), which may be a potential coping strategy for efferocytosis 27-29. It has been discovered the activation of LXR accelerated the clearance of apoptotic cells and the resolution of lung inflammation 30, 31. The dual needs to maintain the availability of phagocytic receptors to be activated and to avoid massive accumulation of cholesterol emphasize the importance of their synergy. Therefore, we expect that their simultaneous administration may greatly potentiate erythrophagocytosis to improve recovery after ICH. Nevertheless, most existing ADAM17 inhibitors and LXR agonists possess poor bioavailability due to low solubility and off-target effect 32-34. Meanwhile, the existence of the blood-brain barrier (BBB) restricts most of therapeutic agents access to the brain 35, 36. How to efficiently and accurately deliver the two above drugs to the damaged situ after ICH becomes the challenge that need to be addressed.

Drug delivery systems (DDS) have been investigated for the treatment of multiple CNS disorders, such as stroke, spinal cord injury, traumatic brain injury, Alzheimer's disease and glioblastoma *etc.* owing to their fascinating advantages including the desirable ability to improve pharmacokinetics of therapeutic drugs, considerable permeability to the BBB and impressive capability to target diseased lesions 37-41. In recent years, nanomaterials based on natural cell membranes have sprouted up as an attractive strategy to biomimetically functionalize nanoplatforms for *in vivo* applications 42. Extractive plasma membranes inherited the surface structure and protein profile of the original cells, and excelled at interacting with complicated biological environments to avoid unexpected immune response 43. More importantly, with cell membrane camouflaging, especially leukocytes membranes, these nanocarriers were endowed with properties of specifically targeting injured sites 44. Neutrophils, the most common white blood cells, could be recruited to the damaged region within hours owing to the interaction between their surface receptor and corresponding ligand overexpressed on stressed vascular endothelial cells (VECs) 45. Therefore, neutrophil membrane camouflaging may be a promising solution to accurately deliver drugs to inflammatory lesions. For example, Feng *et al.* reported neutrophil membrane-coated nanoparticles (NPs) realized efficient navigation to the brain to alleviate the brain damage and improve neurological functional recovery 46. Xiong *et al.* developed a nanoplatform cloaked with a neutrophil membrane for targeting delivery of multifunctional nanozymes to the extrinsic neural environment where reactive oxygen species (ROS) was scavenged and inflammation was reduced to facilitate nerve regeneration 47. However, the non-specific release of drugs also limits the work efficiency of DDS to some extent. The advance of smart DDS, which could release drugs responsive to a variety of internal and external stimuli at the site of injury, has highly removed the roadblock 48-51. Responsive nanomaterials could be developed based on that neuroinflammation, featured by low pH, overexpressed ROS and so forth, is one of the main characteristics after brain disorders 52-54. For instance, Liu *et al.* prepared ROS-responsive phenylboronic dendrimer-peptide conjugates for delivering therapeutic peptides to modulate the inflammatory microenvironment of Alzheimer's disease 55. Yang *et al.* developed a pentapeptides (PHSRN)-conjugated hydroxyethyl starch (HES) nanoplatform for that therapeutic drugs coupled with PHSRN-HES were released under the acidic microenvironment of neuroinflammation 56. He* et al.* designed a 4T1 cell membrane-camouflaged succinobucol-loaded pH-sensitive polymer of methoxy poly (ethylene glycol)-block-poly (2-diisopropyl methacrylate) to responsively release succinobucol in an acidic environment to exert its antioxidant and anti-inflammatory activities 57.

Herein we designed and synthesized a pH-responsive lipid and neutrophil membrane hybrid nanoparticle to synergically deliver hydrophobic desmosterol (an agonist of LXR) and GW280264X (an ADAM17 inhibitor) to inflammatory hemorrhagic sites. The neutrophil membrane granted nanoparticles with chemotactic abilities to orient inflammatory lesions and the incorporated pH-sensitive lipid (DSPE-PEOz) enabled the acceleration of drug release in an acidic microenvironment. Therapeutic investigation revealed that the synergic delivery of GW280264X and desmosterol significantly increased erythrophagocytosis and inhibited inflammatory response. This nano-system could be a promising strategy to effectively promote hematoma and inflammation resolution, consequently alleviate ICH progression.

## Methods

### Materials and Chemicals

Soybean phospholipid was purchased from Shanghai Yuanye Bio-Technology Co., Ltd. 1,2-distearoyl-sn-glycero-3-phosphoethanolamine-poly(2-ethyl-2-oxazoline) (DSPE-PEOz, Mw = 2000 Da) and 1,2-distearoyl-sn-glycero-3-phosphoethanolamine-polyethylene glycol (DSPE-PEG, Mw = 2000 Da) were purchased from Xi'an Ruixi Biological Technology Co., Ltd. Cholesterol was purchased from Aladdin, China. 1,1'-dioctadecyltetramethyl indotricarbocyanine iodide (DiR) was ordered from Yeasen Biotech Co., Ltd, Shanghai, China. 4,6-diamidino-2-phenylindole (DAPI), Histopaque 1119, Histopaque 1077, Ficoll and Drabkin's reagent were obtained from Sigma-Aldrich, USA. Fetal bovine serum (FBS), Dulbecco's modified Eagle's medium (DMEM) and penicillin/streptomycin were purchased from Gibco, USA. Protease inhibitors cocktails, Pierce BCA Protein Assay Kit and RBC Lysis Buffer, Lysotracker were purchased from Thermo Fisher Scientific, USA. 1,1'-dioctadecyl-3,3,3',3'-tetramethylindocarbocyanine perchlorate (DiI), 1'-dioctadecyl-3,3,3',3'-Tetramethylindodicarbocyanine,4-Chlorobenzenesulfonate salt (DiD) and Hoechst 33342 were bought from Beyotime Biotechnology, China. Lectin (FL-1201) was bought from Vector Laboratories, USA. Alexa Fluro 488-conjugated donkey anti-rabbit IgG (A21206), Alexa Fluro 488-conjugated donkey anti-goat IgG (A32814), Alexa Fluro 555-conjugated donkey anti-rabbit IgG (A32794), Alexa Fluro 568-conjugated donkey anti-goat IgG (A11057) were purchased from Invitrogen, USA. IL-10, TNF-α, IL-β, MERTK and AXL ELISA Kits were purchased from Boster Biological Technology, China. SensoLyte 520 TACE (α-Secretase) Activity Assay Kit was purchased from AnaSpec, Inc. company, USA. The OxyHb was obtained from Solarbio, China. Unless otherwise noted, organic solvents and inorganic salts were purchased from Sinopharm Chemical Reagent Co., Ltd., China, and used without further purification. Water in all the experiments was purified using a Millipore-Q water-purification System (Milli-Q Integral 3, Millipore, USA).

### Neutrophil collection and cell membrane derivation

Neutrophils were harvested according to a published protocol 58. In brief, femurs and tibias were collected after C57BL/6 mice were euthanized. The bone marrow cells were flushed with the RPMI 1640 medium into a 50 mL centrifuge tube to generate the single cell suspension, filtered with a 70 µM cell strainer. The liquid samples were centrifuged at 1400 rpm for 7 min (4 °C), followed by resuspending the cell pellet with RBC Lysis Buffer to remove erythrocytes. The neutrophils were isolated from the mixed Histopaque 1119/1077 solution by the density gradient centrifugation at 2000 rpm for 30 min at 25°C. The purified neutrophils were washed and then collected for the following membrane derivation.

Cell membrane derivation was collected following previous reports 59-62. Cells were resuspended with hypotonic lysis buffer containing protease inhibitors and then submitted to five-cycle freezing in liquid nitrogen and thawing at room temperature before transferred to Dounce homogenizer. The homogenized solution was centrifuged (1000 g for 10 min at 4 °C) to remove nuclei and broken cells, followed by centrifugation of the supernatant again at 10000 g for 25 min, 4 °C to remove organelles. Neutrophil membranes were obtained after centrifugation at 100000 g for 1 h at 4 °C, quantified using a BCA kit and then suspended with 0.2 mM EDTA for subsequent studies.

### Synthesis of nanoparticles and characterization of hybrid liposomes

Liposomes were prepared using the thin lipid film hydration method 48. Briefly, 35 mg of soybean phospholipid, 5 mg of cholesterol, and 10 mg of DSPE-PEOz were dissolved in 10 mL of chloroform. Then, 2 mL of the above solution and 8 mL of chloroform were added into a round flask and slowly evaporated to form a thin film by rotary evaporator at 40 °C. Thereafter, 1 mL of sterile PBS (pH 7.4, 10 mM) was added to the round flask and sonicated for 20 min in an ice water bath to obtain pH-responsive liposomes (PEOz). The non-responsive liposomes (PEG) were prepared using DSPE-PEG instead of DSPE-PEOz. Drug-loaded liposomes were obtained using the same method, except that drugs were dissolved in chloroform with a lipid component with an initial desmosterol-lipid ratio of 1:30 (w/w) (D@PEOz), GW280264X-lipid ratio of 1.5:30 (w/w) (G@PEOz), or desmosterol-GW280264X-lipid ratio of 1:1.5:30 (w/w/w) (D&G@PEOz or D&G@PEG). The fluorochrome DiR-loaded liposomes were obtained using an initial DiR-lipid ratio of 0.3:30 (w/w). Neutrophil cell membrane hybrid liposomes (D&G@NPEOz or D&GNPEG) were gained via an extruder set (Avanti Polar Lipids, Inc., USA). Different proportion of liposomes and proteins on neutrophil cell membranes (30:1 or 20:1 w/w) were mixed and subsequently sonicated in an ice water bath for 5 min. The mixtures were extruded in order 10 times at 42 °C through 200, 100 and 50 nm polycarbonate porous membrane filters using an extruder.

The hydrodynamic diameter and zeta potential of all liposomes were determined by dynamic light scattering (DLS, Zetasizer 3000, Malvern, USA). Measurement was repeated three times to obtain an average value. The morphologies were observed under transmission electron microscope (TEM, HT-7700, Hitachi, Japan) at 200 kV after placing a drop of the liposome solution on a copper grid coated with amorphous carbon and staining with phosphotungstic acid solution (2%). Furthermore, the component of hybrid liposomes was characterized by the functional proteins via western blot (WB) 46, 63.

### Drug loading capacity, stability, and pH-responsive drug release manner of hybrid liposomes

After preparing the liposomes using an extruder, any unencapsulated free drugs were removed via ultrafiltration tubes (Millipore, MWCO = 3500 Da). Then, the encapsulated drugs were collected by extracting the liposomes with chloroform, and the desmosterol was tested with high performance liquid chromatography (HPLC, 1260Ⅱ, Agilent Technologies, USA) while GW280264X was measured by ultraviolet spectrometer (UV, UV2600, Shimadzu, Japan). Standard curves of drugs were obtained at the same time. A reverse-phase column (C18, 150 × 4.6 mm, 3.5 μm) and an ultraviolet detector (236 nm) were used. The mobile phase was made of methanol: acetonitrile = 50:50 (v/v), with a flow rate of 1 mL/min and an injection volume of 20 µL. The column temperature of 30 °C was employed. Liposomes at a concentration of 1 mg/mL in water, 10 mM pH 7.4 PBS, or DMEM were stored at 4 °C. After 6, 24, 48 and 72 h of storage, the hydrodynamic diameters of liposomes were detected by DLS. Next, the pH-responsive drug release of the liposomes was investigated. Specifically, D&G@NPEOz were packed in a dialysis tubing (MWCO = 3500 Da) and placed in 5 mL PBS (pH 5.5, 6.5 or 7.4) at 37 °C water bath with orbital shaking at 200 rpm. At predetermined time intervals, the PBS was removed followed by the addition of another 5 mL of PBS with the same pH. The drug release amount was determined by HPLC and UV.

### Cytotoxicity and cell uptake *in vitro*

The cytotoxicity of D&G@NPEOz nanoparticles against BV2 cells was evaluated by CCK-8 assay. In brief, the microglial cell line BV2 cells from the American Type Culture Collection (ATCC) were cultured in DMEM added with 10% FBS plus penicillin (100 U/mL) and streptomycin (100 µg/mL) in 37°C incubators with 5% CO_2_. BV2 cells were plated in a 96-well plate (10000 cells/well) and incubated overnight and then the culture media were substituted with the fresh medium containing D&G@NPEOz at different concentrations. After incubation for 24 h, the culture media were replaced by FBS-free medium containing 10% CCK-8 for another co-incubation for 1 h. The absorbance at 450 nm was then determined using a microplate reader.

DiD dye-labeled nanoparticles were used for cell uptake assay. BV2 cells were seeded in a 6-well plate (1.0 × 10^6^ cells/well) overnight firstly. 50 µg/mL nanoparticles were added and incubated with cells for 2, 4, and 6 h at 37°C. The cells were washed three times with PBS and collected after incubation. The mean fluorescence intensity (MFI) determined by FACS represented cellular uptake. For confocal images, BV2 cells were plated in cover-glass-bottom confocal dishes at a density of 5.0 × 10^5^/dish overnight in a 37°C incubator. The same treatment was performed as mentioned above. Instead of harvesting cells, the cells were stained with Lysotracker and Hoechst 33342 respectively according to their manufacturer's instructions and then observed using laser scanning confocal microscopy (LSCM).

### Erythrophagocytosis assay *in vitro*

Red blood cells (RBCs) were isolated from murine whole blood by means of Ficoll gradient centrifugation and then washed twice in PBS. The lipophilic fluorescent probe DiI or DiD was utilized to generate fluorescent erythrocytes as per the manufacturer's instructions. To induce apoptosis, the labeled RBCs were incubated in a water bath (56 °C) for 5 minutes 2. For FACS assay, BV2 cells with different NPs treatments were co-cultured with DiD-labeled apoptotic RBCs (5-10 times the numbers of phagocytes) at 37 °C for 2 h. Free RBCs were removed via RBC Lysis Buffer according to the manufacturer's procedures, and then washed three times with PBS before the samples were subjected to FACS to analyze erythrophagocytosis. For immunofluorescence imaging, DiI-labeled apoptotic erythrocytes were added to BV2 cells which had been plated on 12-well plates with coverslips and treated with different NPs. After extracellular RBCs were removed, the cells were fixed with paraformaldehyde and then mounted with Lectin and DAPI on a glass slide. Erythrophagocytosis was observed with the help of Lazer Scanning Confocal Microscope (LSCM).

### Animals

All experiments involving animals were performed in accordance with the guidelines of animal care and use and authorized by the ethics committee of the Second Affiliated Hospital (Zhejiang University, China, Approval No. AIRB-2021-607). Male C57BL/6 mice used in our experiments were housed in a humidity and temperature-controlled animal faculty with a 12 h light/dark cycle.

### ICH model

ICH model was established by injecting donor blood in adult male mice (25-30 g) as previously described 5. Briefly, the mice were firstly induced to anesthesia with 4% isoflurane inhalation, and then were continuously anesthetized via a mask connected to 1.5% to 2% isoflurane during the operation. For ICH model, 30 µL donor blood was collected and injected to striatum at the following coordinates (2.5 mm lateral and 3.0 mm deep at a 5° angle relatively to bregma). Bone wax was used to seal the craniotomy and 3M Vetbond tissue adhesive was applied to close the scalp. The body temperature of mice was maintained at 37.0 ± 0.5 °C with the help of a temperature-controlled heating pad. ICH mice were intravenously injected with 100 µL different particles once a day.

### Brain targeting ability

The ICH mice were intravenously immediately injected with free DiR, DiR@PEOz, DiR@NPEOz at 2 mg/kg, as referenced to a previously published literature 64. Mice were observed using an *in vivo* imaging system at different times. The mice were sacrificed after 12 h and the brain, heart, liver, lung, kidney, and spleen were collected. All the organs were imaged using an *in vivo* imaging system immediately.

### Erythrophagocytosis assay *in vivo*

DiD-labeled RBCs were resuspended in autologous plasma with 1:4 ratio as described previously 2. The recombined-fluorescent blood was used to induce the ICH model 5. Mice were sacrificed at 3 days after ICH induction and the brain samples were analyzed by flow cytometry. The RBCs-engulfing microglia/macrophages were defined as the LIVE/DEAD^-^CD45^int/high^CD11b^+^ DiD-RBCs^+^ population.

### Behavioral tests

### Two researchers blinded to the experiment state performed the neurobehavioral function test

Cylinder test. Mice were placed in a transparent glass cylinder where they were allowed to rear and place their forelimb paws on the wall of the cylinder freely. We recorded the first forelimb (right, left, or both) placed the cylinder wall for a total of 20 times. The laterality index was identified as (right - left)/ (right + left + both). Obviously, injured mice exhibit a preference for using of their right forepaws (ipsilateral to lesion) relying on the severity of the injury whereas healthy mice display no preference for either forepaw.

Corner turn test. The mice were allowed to turn either left or right to exit a 30° transparent corner of two plastic walls freely. A total of 10 trials were recorded to assess the sensory and motor function balance. The direction of preference was reflected by the percentage of right turns.

Forelimb placement tests. The bodies of mice were held and their vibrissae were brushed on the edge of a countertop. Ten measurements were performed on each side. Uninjured mice could rapidly place the ipsilateral forelimb after stimulation. The percentage of successful placements was calculated to evaluate the severity of the injury.

### Residual hematoma volume measurement

Mice were sacrificed and perfused transcardially with the cold PBS at day 3 after ICH. The 1-mm-thick coronal sections across the hematoma region of the brains were obtained according to a previous procedure. The residual hematoma volume was analyzed and quantified with ImageJ software (NIH) after the digital images of brain slices were acquired. The total hemorrhage volume was calculated in cube milliliters by summing the hemorrhage area of each section multiplied the thickness of each section (1 mm). Residual hemoglobin content assay with Drabkin's reagent was also conducted to estimate cerebral hematoma. Briefly, coronal brain slices containing blood were collected and homogenized in 300 µL doubled instilled water, followed by sonication to lyse the cells. Supernatants were harvested after samples were centrifuged with 15000 g for 30 min. 20 µL supernatants were incubated with Drabkin's reagent (80 µL) for 15 min at room temperature. Optical density values were measured by spectrophotometry at a wavelength of 540 nm and then the concentration of cyanmethemoglobin was assessed based on a standard curve which was generated according to the following procedure: incremental volumes of the blood (0, 0.5, 1.0, 2.0, 4.0, and 8.0 µL whole blood) were added into lysate specimens from normal brains and the optical density was simultaneously measured.

### Immunofluorescence staining

Immunofluorescence staining was performed on frozen coronal sections obtained from harvested mice brains. In reference to the protocol, the coronal sections were washed and then blocked with 10% donkey serum containing 0.1% Triton X-100. Then the sections were incubated overnight at 4 °C with primary antibodies including Iba-1 (ab5076, 1:500, Abcam), iNOS (18985-1-AP,1:500, Proteintech), CD206 (ab64693, 1:500, Abcam). The sections were then labeled with corresponding secondary antibodies and incubated for 2 h at 37 °C after washed with PBS several times. Finally, the acquired slices were washed again, mounted with DAPI, and then observed under a fluorescent microscope (Leica, Mannheim, Germany).

### ELISA

Protein extracts from perihematomal brain tissue were collected and quantified by BCA kit. Plasma samples from ICH mice and cell culture medium after different NPs treatments were also harvested for ELISA analysis. The levels of sMerTK, sAXL, IL-10, TNF-α, and IL-β in the samples were determined with the ELISA kit according to the manufacture's protocol. All samples and standards were analyzed in duplicate.

### Western blot

Proteins of brains or cells were prepared according to the standard procedures 65. In brief, brain tissues surrounding the hematoma or cultured cells were extracted with cold RIPA lysis buffer. The protein content was determined using a Pierce BCA Protein Assay Kit. Equal amounts of protein from each sample were loaded onto PAGE-SDS gels, separated by electrophorese and then transferred to polyvinylidene difluoride (PVDF) membranes. Afterwards the membranes were blocked with 5% milk in the Tris buffered saline with Tween 20 (TBST) for 1 h at room temperature before being incubated with the following primary antibodies: anti-ABCA-1 (1:500, PA5-86023, Thermo Fisher Scientific), anti-ADAM17 (1:1000, 29948-1-AP, Proteintech), anti-ABCG-1 (1:1000, 13578-1-AP, Proteintech), anti-β-Actin (1:5000, ab8226, Abcam) overnight at 4°C. After several washes with TBST, the membranes were then processed with appropriate HRP-conjugated secondary antibodies (1:10000) for 1 h at room temperature. Blot bands were observed via chemiluminescence (ECL).

### ADAM17 activity assay

Proteins from brains and BV2 cells were lysed in activity assay buffer (pH 7.4, 25 mM NaCl, 50 mM Tris HCl, 10 mM ZnCl_2_ and 4% glycerol) after different NPs treatments. ADAM17 activity was measured by using the SensoLyte 520 TACE (α-Secretase) Activity Assay Kit in accordance with the manufacturer's instructions.

### Biosafety *in vivo*

Healthy mice were treated with PBS and D&G@NPEOz through intravenous injection. At day 1 and day 7 whole blood and main organs including the brain, heart, liver, spleen, lung and kidney were collected. The levels of alanine aminotransferase (ALT), aspartate aminotransferase (AST), creatinine (CRE) and urea nitrogen (BUN) were determined as well as white blood cells (WBCs), neutrophil (Neu), red blood cell (RBC), platelets (PLT) count according to the manufacture's instruction. In the meantime, hematoxylin-eosin (H&E) staining was applied to assess any changes of the main organs.

### Statistical analysis

Quantitative data are represented as mean ± standard deviation (SD). Statistical analysis was performed with GraphPad Prism 8.0 software (GraphPad Software Inc., La Jolla, CA, USA). A one-way analysis of variance (ANOVA) followed by the Tukey's post hoc test was carried out to test normal distribution and homogeneity of variance and compare the difference between multiple groups. The Kruskal-Wallis test with Bonferroni correction for post hoc test comparisons was used to compare the non-normal distribution and unequal variance parameters. Two-way ANOVA followed by the Tukey's post hoc test was used to analyze the persistent neurological functions. Statistical significance was considered at *P* < 0.05.

## Results and Discussion

### Combinational effect of drugs

**Figure 1:**
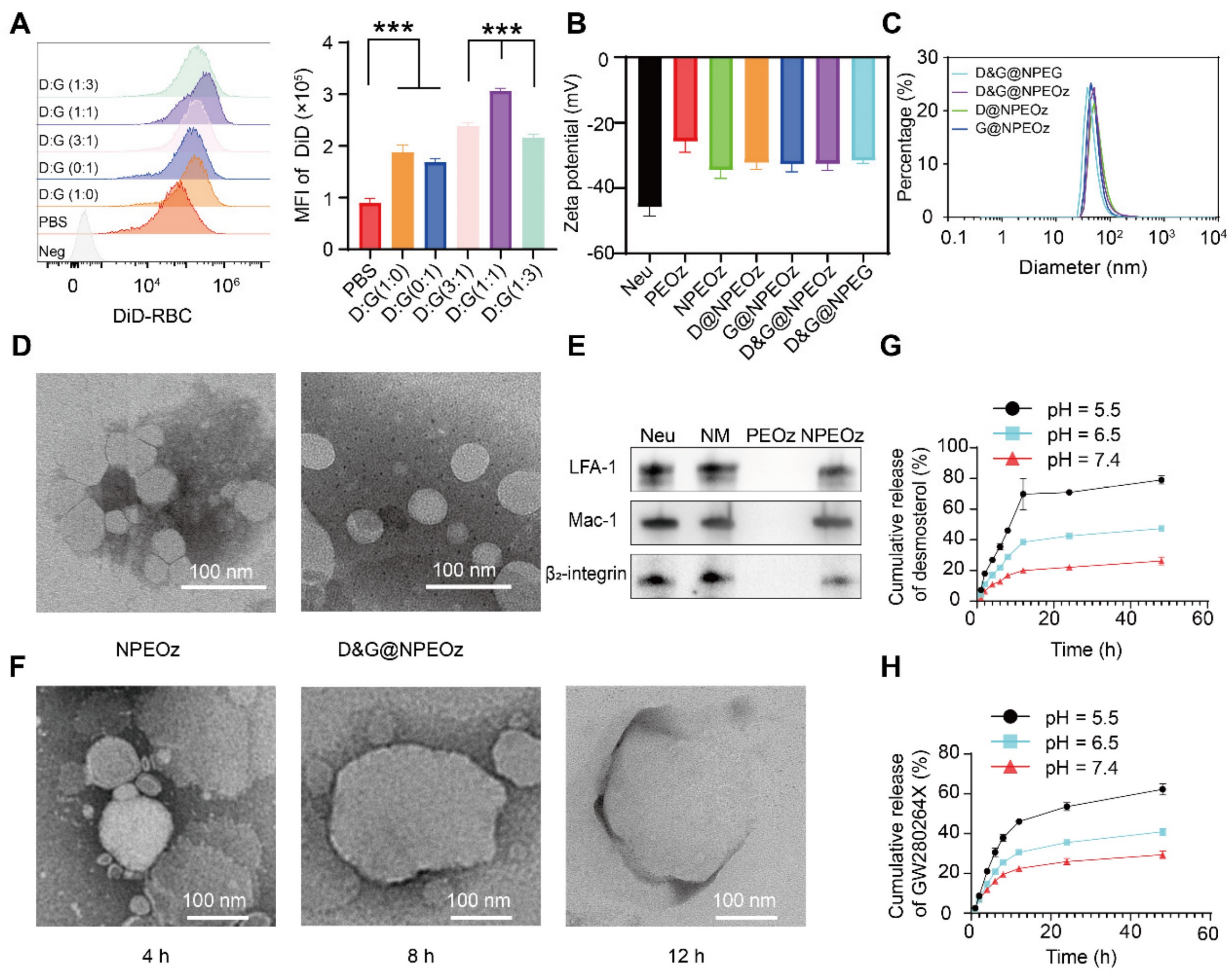
** The characterization of D&G@NPEOz.** (**A**) Representative flow cytometry histogram and quantity analysis of mean fluorescence intensity (MFI) of engulfed labeled-erythrocytes in BV2 cells with different radios of desmosterol and GW280264X treatment followed by 2 h incubation with DiD-labeled RBCs (n = 3). (**B**) Zeta potential of NPEOz, D&G@NPEG, D&G@NPEOz, D@NPEOz, G@NPEOz (n = 3). (**C**) Hydrodynamic diameters distribution of D&G@NPEG, D&G@NPEOz, D@NPEOz, G@NPEOz. (**D**) TEM images of NPEOz and D&G@NPEOz (Scale bar = 100 nm). (**E**) Representative western blot showing characteristic protein expression from Neu (neutrophil), NM (neutrophil membrane), PEOz and NPEOz. (**F**) The morphological changes of TEM images of D&G@NPEOz with time while incubated in PBS at pH 5.5 (Scale bar = 100 nm). (**G-H**) Desmosterol and GW280264X release characteristics of D&W @NPEOz in PBS at pH = 5.5, 6.5 or 7.4 during 48 h incubation (n = 3). All data are presented as means ± SD. * versus indicated groups, *** *P* < 0.001.

The befitting concentration and combination ratio of desmosterol and GW280264X were firstly determined. A murine microglia-derived cell line, namely BV2, was chosen used in studies *in vitro* due to that it largely possesses the functional, phenotypic and morphological characteristics of microglia 66. BV2 cells were firstly treated with desmosterol and GW280264X at varying concentrations ranging from 1 μM to 20 μM to determine the optimal concentration that promotes erythrophagocytosis of BV2 cells. Flow cytometer results showed that the effect of both desmosterol and GW280264X on erythrophagocytosis of BV2 cells was concentration-dependent. Both desmosterol and GW280264X displayed relatively excellent effect on erythrophagocytosis of BV2 cells at 10 μM (**Figure S1-2**). Therefore, their concentrations at 10 μM were selected for further experiments. Then varied combination ratios of the two drugs were employed to evaluate their synergistic effect. By using desmosterol/GW280264X at molar ratio of 1:0, 3:1, 1:1, 1:3 and 0:1, it was found that the molar ratio of desmosterol and GW280264X at 1:1 exerted the strongest fluorescence intensity which meant optimal erythrophagocytosis (**Figure 1A**). Based on this result, we chose the molar ratio of 1:1 for desmosterol and GW280264X to synthesize D&G@NPEOz.

### Physiochemical properties of D&G@NPEOz

**Scheme 1:**
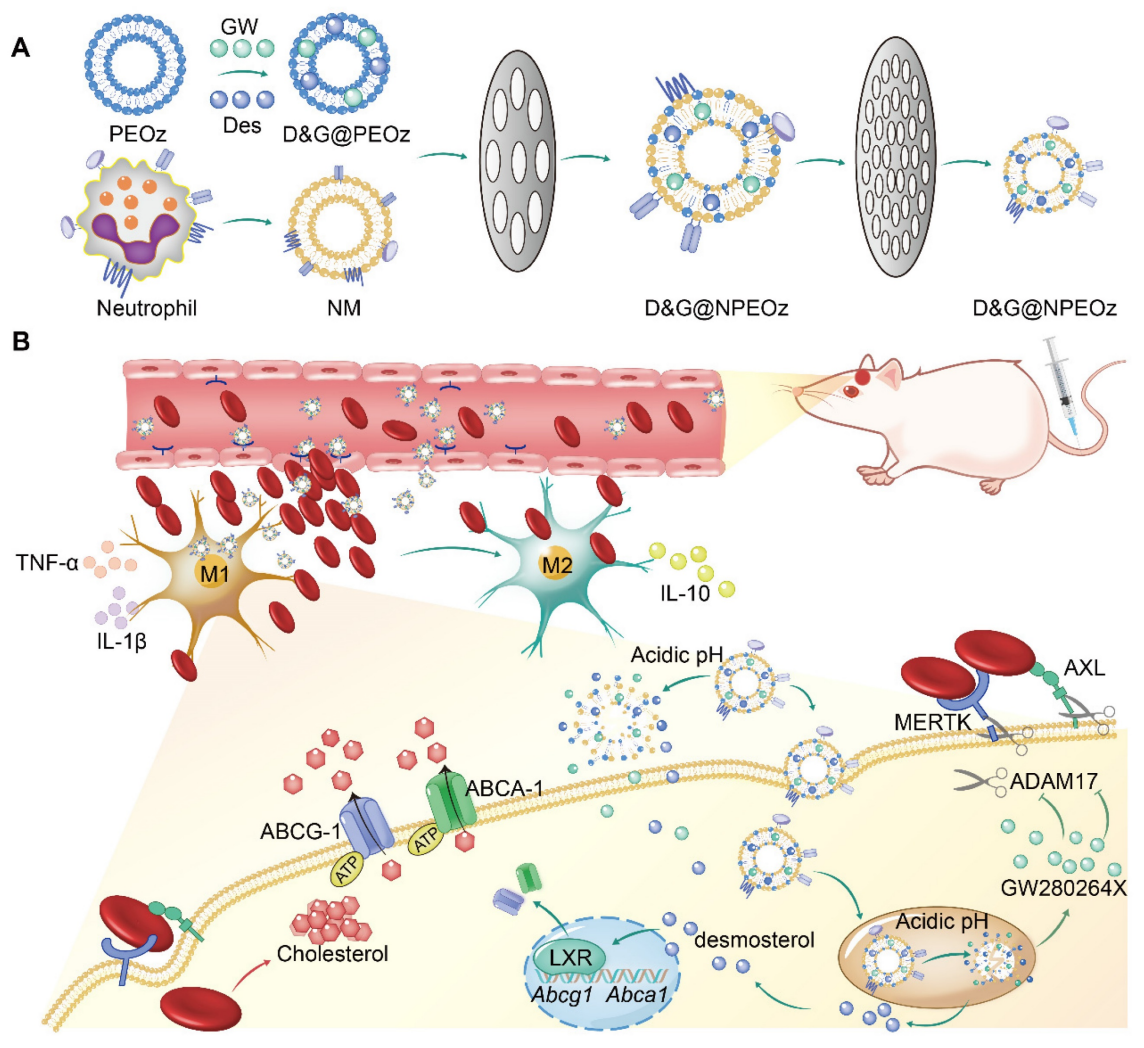
Illustration of D&G@NPEOz therapy for ICH. (**A**) Scheme of the D&G@NPEOz synthesis process. (**B**) Illustration of D&G@NPEOz therapy for erythrophagocytosis and neurological functional recovery after ICH. Camouflaging of neutrophil membrane efficiently deliver nanoparticles to insult site across brain microvascular endothelial cells. The D&G@NPEOz releases desmosterol and GW280264X responsive to the acid environment to promote erythrophagocytosis by microglia/macrophage through activation of LXR and inhibition of ADAM17, then drive microglia/ macrophage polarization from M1 to M2 and inhibit inflammation, consequently elicit neurological recovery after ICH.

The preparation of D&G@NPEOz involved the extraction of neutrophil membranes which contain surface chemotactic proteins from the neutrophils and their fusion with synthetic functional liposomes, as illustrated in **Scheme 1.** Neutrophils were firstly isolated from the bone marrow of C57BL/6 mice and purified through the Histopaque 1119/1077 mixture gradient centrifugation method. Flow cytometer results showed the purity of the isolated neutrophils was approximately 90% (**Figure S3**). The cell membrane derivation from these harvested neutrophils was further collected through hypotonic ultracentrifugation, and the protein content of cell membrane derivation was quantified using a BCA kit. The protein content in the cell membrane production derived from 300 million cells was found to be about 1 mg. The liposomes were prepared using the filming-rehydration method and then fused with neutrophil cell membrane via co-extrusion to obtain hybrid liposomes. By selecting 50 nm as the final pore size of polycarbonate filters, the diameters of all hybrid liposomes were maintained approximately 50 nm with a narrow particle size distribution measured by dynamic light scattering (DLS) (**Figure 1C**). Meanwhile, the typical transmission electron microscopy (TEM) images confirmed that both NPEOz and D&G@NPEOz exhibited uniform spherical vehicles, consistent with the data obtained from DLS measurements (**Figure 1D**). The zeta potentials of these hybrid liposomes in solution were around -30 mV (**Figure 1B**).

Neutrophils could rapidly appear in inflammatory sites, which is attributed to the key role of their specific membrane proteins involved in the course of lesions targeting. In order to determine whether nanoparticles inherited the main targeting proteins from neutrophils, western blot analysis was conducted to investigate the changes of membrane proteins before and after co-extrusion. The results demonstrated that extracted neutrophil membranes retained most of the critical chemotactic proteins present on neutrophils (**Figure 1E**), including LFA-1, Mac-1 and β2-integrin which are known to involve in targeting progress by interacting with corresponding ligands like intercellular adhesion molecule-1 (ICAM-1) that overexpressed on the lumen side of irritative VECs in insult sites 46. More importantly, the key proteins responsible for promoting neutrophils infiltration into lesions were successfully translocated onto nanoplatforms through membrane extrusion (**Figure 1E**), confirming the successful fusion of liposomes and neutrophil membranes and the potential neutrophil-like chemotactic feature of these nanocarriers.

To further optimize the encapsulation efficiency of two drugs into PEOz, we altered the feeding ratios of drugs and PEOz. As shown in **Figure S4**, in the case of maintaining the molar ratio of desmosterol and GW280264X at 1:1, the adjustment did not significantly affect the encapsulation efficiency while the feeding ratio of drugs and PEOz varied from 1:96 to 1:12. However, when the feeding ratio of drugs and PEOz reached 1:6, the encapsulation efficiency dropped precipitously. Therefore, the corresponding formulation (weight ratio of desmosterol: GW280264X: PEOz = 1:1.5:30) was used for subsequent experiments. Under the circumstances, the encapsulation efficiencies of the desmosterol and GW280264X in liposomes were found to be 51.4% and 79.8%, respectively. Afterwards, we investigated the stability of D&G@NPEOz in water, PBS (pH 7.4) and DMEM for three days. Little changes in size were observed (**Figure S5**), indicating excellent stability of the prepared nanocarrier under physiological conditions.

Hydrophobic PEOz segments would become hydrophilic when protonated under acidic conditions 67, which may then trigger liposomes disintegration and release of the drugs. Therefore, we next studied the pH-sensitive functionality of D&G@NPEOz to confirm its capacity of pH-triggered cargo release. The drug release manner of D&G@NPEOz at pH 7.4 was slower than that at pH 6.5 and 5.5, with both reaching a plateau at 12 h, which indicated nanoparticles could release drugs in extracellular acidic inflammatory microenvironment and intracellular lysosome. In particular, 26.2% desmosterol and 29.3% GW280264X were released after 48 h in pH 7.4 solution, 47.4% desmosterol and 40.9% GW280264X were released after 48 h in pH 6.5 solution, while 79.1% desmosterol and 62.2% GW280264X were released after 48 h in pH 5.5 solution, respectively (**Figure 1G-H**). Furthermore, morphological investigation of D&G@NPEOz under acidic conditions using TEM imaging showed a gradual increase in particle size, which was due to the hydrophilic transformation of PEOz segment, a process that disrupted the vesicle structure and made nanoparticles unstable and agglomerate (**Figure 1F**). Such phenomenon was consistent with Liu's report 67.

### Cytotoxicity and uptake of nanoparticles *in vitro*

**Figure 2:**
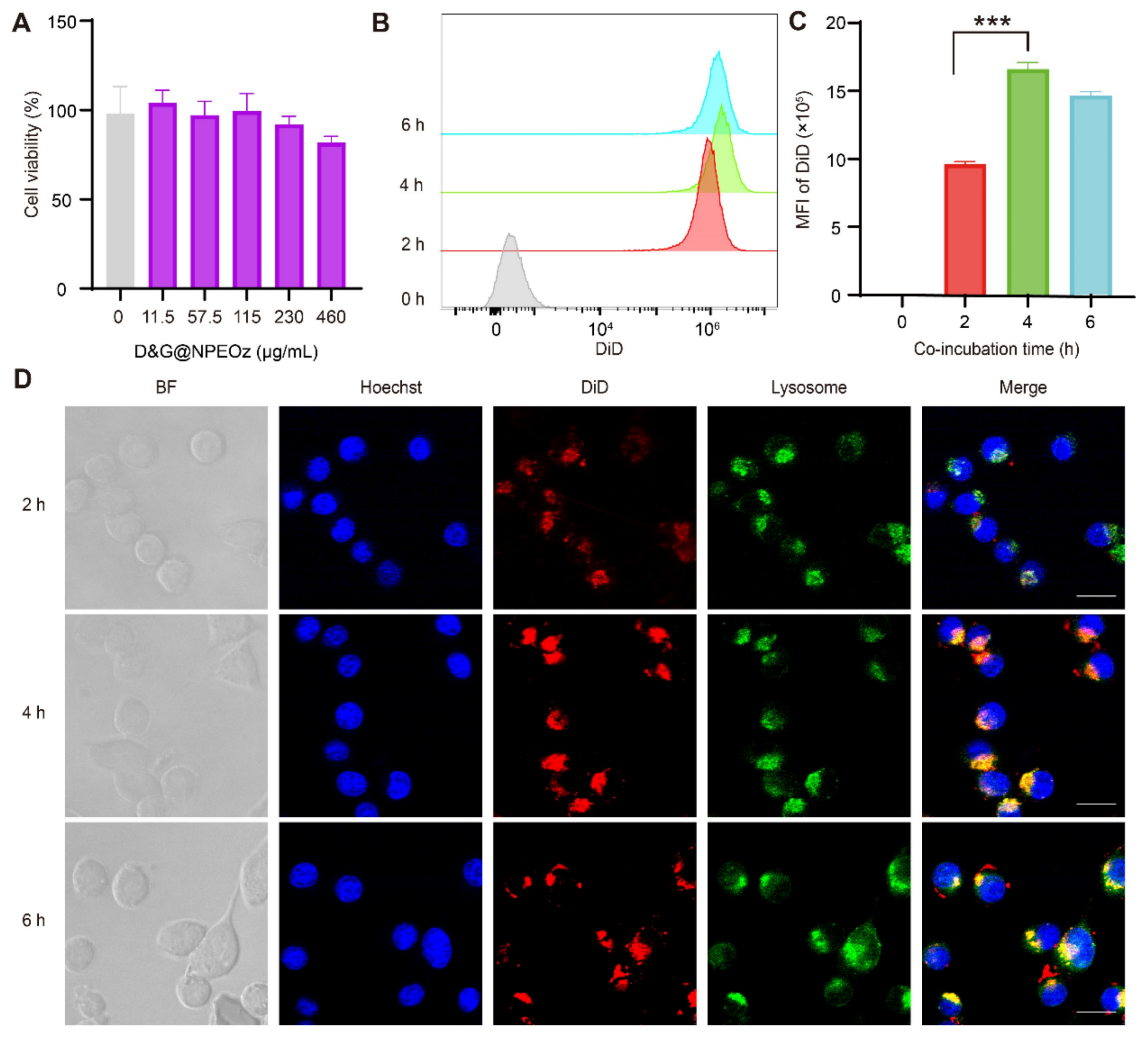
**Biocompatibility of D&G@NPEOz and cellular uptake of BV2 cells.** (**A**) Cell viability of BV2 cells incubated with different concentrations of D&G@NPEOz for 24 h (n = 4). (**B-C**) Typical flow cytometry histogram and quantified analysis of cellular uptake by BV2 cells after incubated with DiD@NPEOz for different time (n = 4). (**D**) Representative confocal images of cellular uptake in BV2 cells incubated with above nanoparticles for different times (Hoechst 33342 in blue, DiD@NPEOz in red, lysosome in green, the gray images represented bright field images. Scale bar = 20 μm). All data are presented as means ± SD. * versus indicated groups, *** *P* < 0.001.

As the most immune surveillance cells in the central nervous system, microglia rapidly respond to stimuli and migrate to lesions to remove exogenous substances and endogenous debris for maintaining the hemostasis 11. We first evaluated the biosafety of nanoparticles *in vitro* before they were used in the further studies. As illustrated in **Figure 2A**, the rate of cell viability measured by the CCK8 kit exhibited at a high level, more than 80% of that observed in the control group, even when incubated with D&G@NPEOz nanoparticles at a concentration of 460 μg/mL after 24 h. This indicated that D&G@NPEOz had good cytocompatibility and safety. Endocytosis of NPs is the first step for the interaction between the loaded drug and the intracellular effector molecules. The cellar uptake assay was performed using DiD-labeled NPEOz (DiD@NPEOz). The results from flow cytometry showed that the cellar uptake of DiD@NPEOz by BV2 cells was time-dependent, with the peak emerging at 4 h (**Figure 2B**-**C**). Consistent with flow cytometry findings, similar results were observed by confocal microscopy. It was worth noting that the internalized DiD@NPEOz undergone endolysosomal trafficking as evidenced by that late endosomes and lysosomes stained by LysoTracker were observed to co-localize with red fluorescence signal from DiD@NPEOz within BV2 cells (**Figure 2D**). Indeed, the late endosome and lysosomes are intracellular acidic compartments where the low-pH environment is perfect to conduce to the decomposition of pH-responsive nanocarriers and the release of loaded drugs.

### D&G@NPEOz enhanced erythrophagocytosis *in vitro*

**Figure 3:**
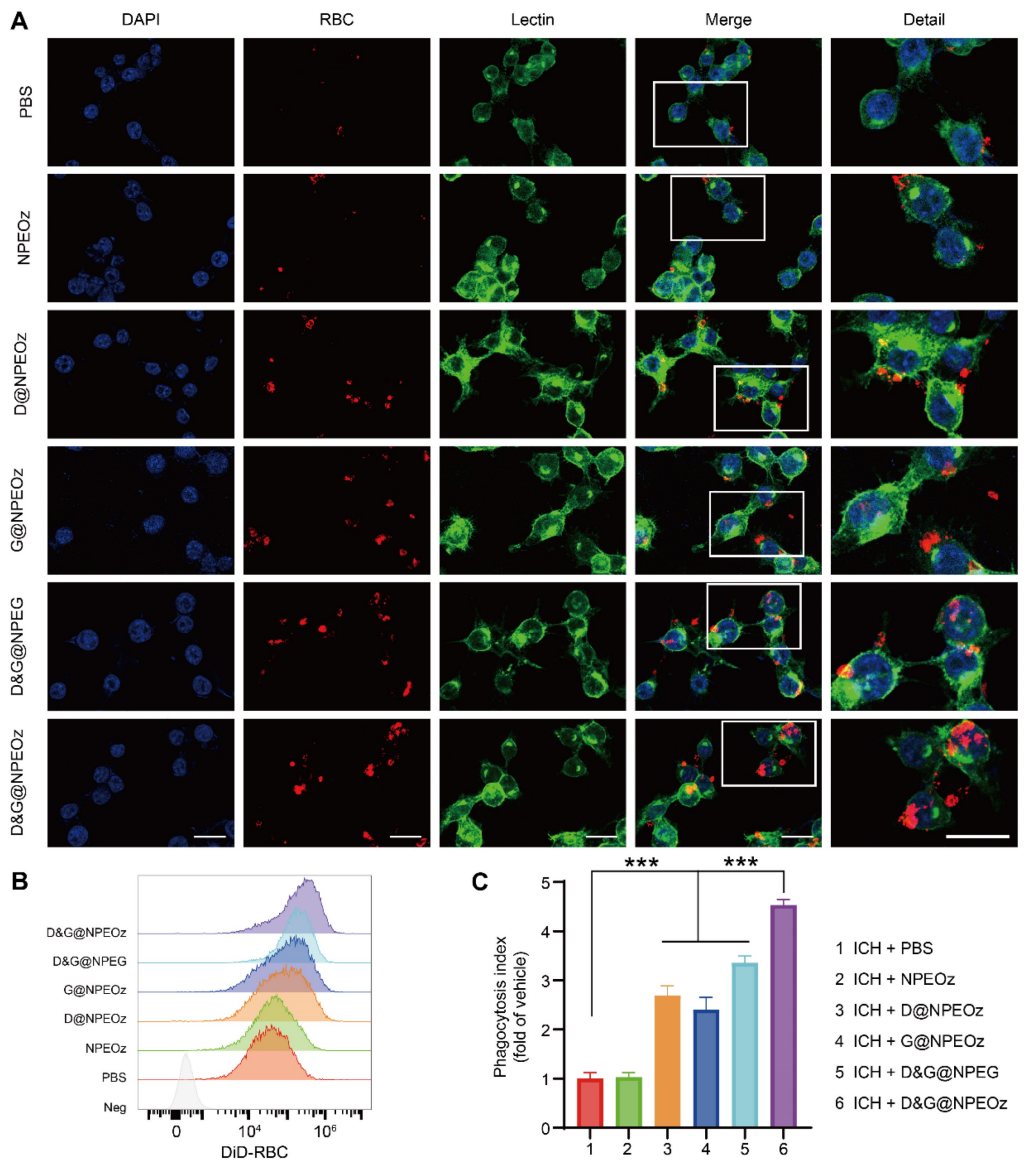
**D&G@NPEOz enhanced efferocytosis.** (**A**) Representative confocal images showing erythrophagocytosis of BV2 cells with different formulations treatment (DAPI in blue, DiI-labeld apoptotic RBC in red, Lectin in green. Scale bar = 20 μm). (**B-C**) Representative flow cytometry histogram showing erythrophagocytosis and quantification of erythrophagocytosis through phagocytic index (calculated as MFI of engulfed fluorescent-labeled erythrocytes in BV2 cells with different formulations treatment relative to PBS treatment, n = 3). All data are presented as means ± SD. * versus indicated groups, *** *P* < 0.001.

Efficacious clearance of apoptotic cells, known as efferocytosis, is crucial to maintain normal physiological states and restore homeostasis after injury 21. Despite lacking nuclei and mitochondria, erythrocytes could be predisposed to become apoptosis-like cells through phosphatidylserine externalization undergoing a process, termed eryptosis, when stimuli impair the integrity of erythrocytes 68, 69. Then apoptotic erythrocytes were removed through erythrophagocytosis. Indeed, erythrophagocytosis after ICH was a form of efferocytosis 11, 70. As mentioned above, we speculated that NPs containing an ADAM17 inhibitor and LXR agonist could more efficiently boost erythrophagocytosis. To confirm our hypothesis, we firstly observed the effect of nanoparticles on erythrophagocytosis *in vitro.* BV2 cells were seeded on a 6-well plate and then suffered from different nano-formulations prior to treatment with 30 μM OxyHb which was used to simulate ICH *in vitro*. After a period of treatment, BV2 cells were incubated with fluorescence-label apoptotic RBCs for 2 h to evaluate the ability of the erythrophagocytosis. According to the results of our pre-experiment (**Figure S6**), the effect of D&G@NPEOz on erythrophagocytosis of BV2 cells was time-dependent and the engulfment of erythrocytes in BV2 cells reached a high level around 12 h after NPs treatment. Therefore, we chose 12 h as the time point for observing phagocytosis ability after nanomedicine treatment. After stimulation for 12 h, It was noticed that BV2 cells exposed to either NPs loading the ADAM17 inhibitor or LXR agonist brought about a slight increase in phagocytosis of apoptotic erythrocytes (**Figure 3A**). It was further validated that there was more erythrophagocytosis observed in BV2s cells co-cultured with D&G@NPEOz NPs, as the fact that Lectin^+^ BV2 engulfed more DiI^+^ apoptotic RBCs, compared with D@NPEOz or G@NPEOz. Of note, pH-responsive nanoparticles enhanced erythrophagocytosis of BV2 cells to a much more notable extent than the non-responsive D&G@NPEG (**Figure 3A**). The similar results were observed by flow cytometry analysis further supported these findings (**Figure 3B**-**C)**, revealing that BV2 cells incubated with D&G@NPEOz have the highest phagocytosis index compared with other NPs (4.5 in D&G@NPEOz group, 2.7 in D @NPEOz group, 2.4 in G@NPEOz group, 3.4 in D&G@NPEG group). Collectively, our results indicated that the pH-responsive D&G@NPEOz was a potent inducer of erythrophagocytosis.

**Figure 4:**
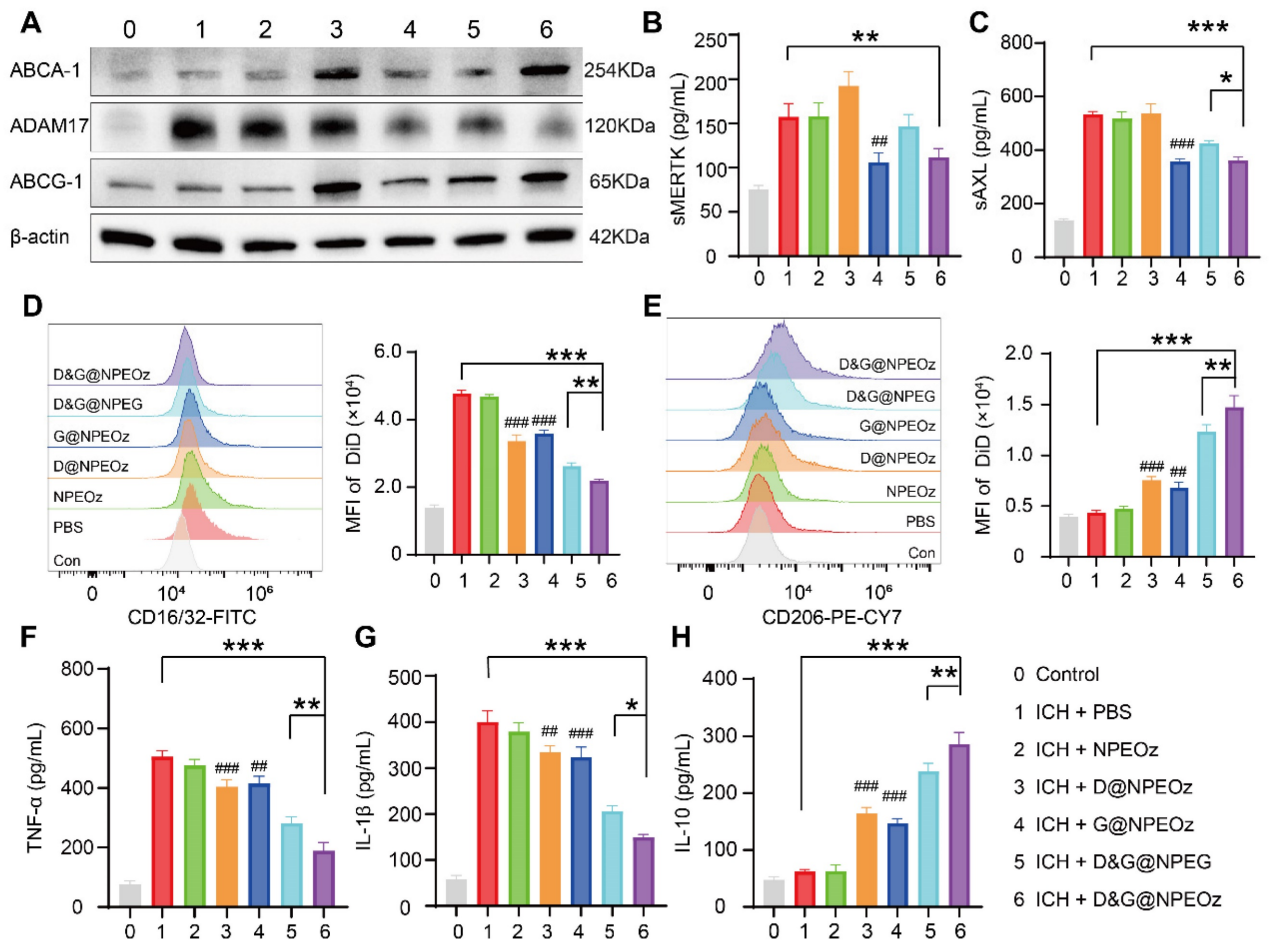
** The mechanisms of pro-efferocytic action and the regulation of proinflammatory microenvironment by D&G@NPEOz NPs *in vitro*.** (**A**) Western blot images showing the expression levels of ABCA-1, ADAM17, ABCG-1 in BV2 cells with different formulations treatment. (**B-C**) The levels of sMERTK and sAXL in the culture media of BV2 cells with different formulations treatment, evaluated using ELISA. (**D**) Representative flow cytometry histogram and quantitative analysis of CD16/32 (a M1 marker) expression on BV2 cells with different formulations treatment (n = 3). (**E**) Representative flow cytometry histogram and quantitative analysis of CD206 (a M2 marker) expression on BV2 cells with different treatment (n = 3). (**F-H**) The levels of TNF-α, IL-β and IL-10 in the culture media of BV2 cells with different formulations treatment, evaluated using ELISA (n = 3). All data are presented as means ± SD. # versus ICH + PBS group, ##*P* < 0.01, ### *P* < 0.001; * versus indicated groups, *P < 0.05, **P < 0.01, *** *P* < 0.001.

To elucidate the mechanisms of enhanced erythrophagocytosis in BV2 cells with D&G@NPEOz treatment, we investigated the levels of soluble MERTK (sMERTK) and AXL (sAXL), protein expression of ADAM17 as well as the activity of ADAM17 *in vitro*. As well reported, MERTK and AXL were pivotal receptors which participated in the efferocytosis of eryptotic erythrocytes through recognizing externalized phosphatidylserine 2. Unfortunately, the cleavage of MERTK/AXL by ADAM17 generated soluble forms (sMERTK and sAXL), which consequently reduced the availability of the cell surface receptors for activation, and impeded the clearance of apoptotic cells 17-20, 23. As shown in **Figure 4A** and** S7**, the protein expression and enzymatic activity of ADAM17 in BV2s pretreatment with G@NPEOz and D&G@NPEOz dramatically decreased. Correspondingly, compared to the PBS group, the level of sMERTK in the culture media of BV2 cells in G@NPEOz or D&G@NPEOz group declined by 32.9% or 32.0% (**Figure 4B**) and the level of sAXL decreased by 32.5% or 29.0% (**Figure 4C**), respectively. The levels of cargoes like cholesterol mounted markedly each time an erythrocyte was engulfed. Excessive accumulation of cholesterol which was not digested after efferocytosis in a timely manner negatively deteriorated the process in turn as occurred in foam macrophage cells 26. Efferocytes dealt with intracellular cholesterol through induction of the transport protein ATP-binding cassette transporters (ABCA1 and ABCG1) via activation of nuclear receptor liver X receptor (LXR) to prevent the overloaded of cholesterol and impairment of efferocytosis 25-29. As an indicator of LXR activity, the protein expression of ABCA1 and ABCG1 were examined. The introduction of LXR agonist desmosterol into the nanoparticles (D@NPEOz and D&G@NPEOz) notably increased the expression of ABCA1 and ABCG1 in BV2 cells (**Figure 4A**). Collectively, the above data suggested that the combined inhibition of ADAM17 and the activation of LXR could enhance erythrophagocytosis in a synergic manner.

### Pro-efferocytosis D&G@NPEOz modulates the inflammatory microenvironment *in vitro*

Recent data have shed light on that microglia could be activated dynamically into two distinct states, namely the classical proinflammatory M1-like phenotype which secretes a variety of proinflammatory cytokines to exacerbate brain damage and the alternative M2-like phenotype which is generally considered anti-inflammatory and conducive to phagocytosis and damage repair 71. Modulating an M1-to-M2 phenotype shift by inhibiting the former and boosting the latter has been reported to mitigate ICH-induced brain injury 8. Interestingly, efferocytosis of apoptotic erythrocyte actively polarized efferocytes towards the alternative M2-like phenotype while contributing to the resolution of inflammation and tissue repair 2, 11, 21. Therefore, we performed a series of assays *in vitro* to confirm the effect of D&G@NPEOz on moderating microglia polarization from an M1 toward an M2 phenotype. BV2 were suffered to different NPs treatments followed by stimulation with 30 μM OxyHb for 12 h and then incubated with apoptotic RBCs. The expression of CD16/32 (M1 marker) and CD206 (M2 marker) were investigated to monitor the status of BV2. It was noted by flow cytometry that ICH resulted in significantly an increased expression of CD16/32 (**Figure 4D**). The mean fluorescence intensity of CD16/32 in D@NPEOz, G@NPEOz, D&G@NPEG or D&G@NPEOz group declined by 29.5%, 24.7%, 45.0%, or 54.1% in comparison with PBS group, respectively** (Figure 4D)**. Although D@NPEOz and G@NPEOz nanoparticle treatments realized a 1.7-fold and 1.6-fold increase in the expression of CD206, the effect of facilitating BV2 cells polarization to M2 phenotype was further enhanced by D&G@NPEOz as evidenced that the MFI of CD206 in D&G@NPEOz group was 2.0 times and 2.2 times stronger than D@NPEOz and G@NPEOz group, respectively (**Figure 4E**). The phenotypic transformation of microglia from M1 to M2 was usually accompanied by changes in secreted cytokines. We next measured the levels of common inflammation-related cytokines in cell culture media after different treatments. Consistently, it was observed that BV2 cells with D&G@NPEOz treatment secreted the fewest proinflammatory cytokines including TNF-α and IL-β (**Figure 4F-G**) while exhibiting the highest level of anti-inflammatory cytokines IL-10 (**Figure 4H**). These results provided compelling evidence that D&G@NPEOz performed best in promoting the formation of an anti-inflammatory microenvironment.

### Targeting capability of nanocarriers to the intracranial inflammatory hemorrhagic sites

**Figure 5:**
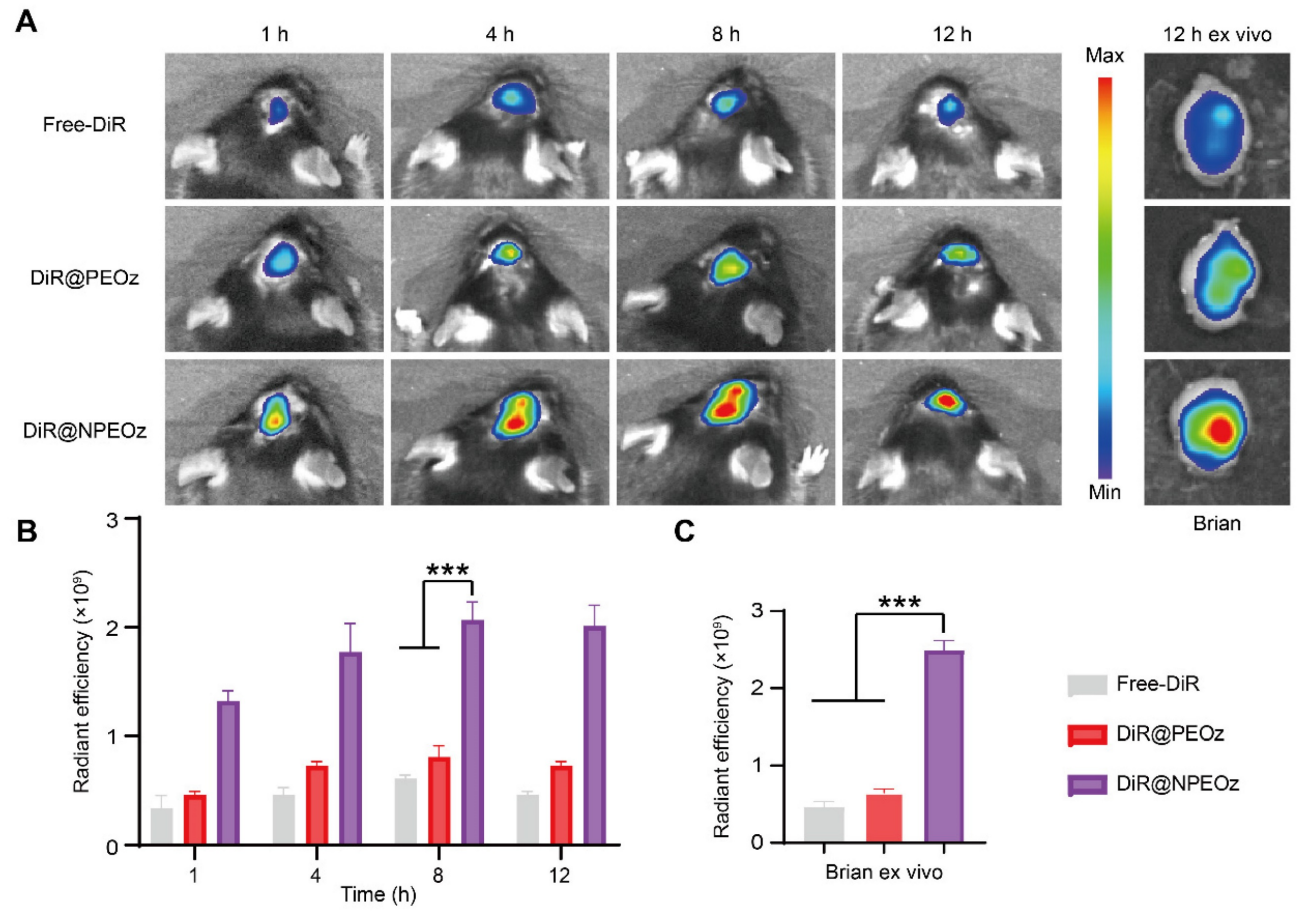
** Neutrophil membrane-mediated targeting ability of nanocarriers.** (**A**) Representative *in vivo* imaging of brain at 1 h, 4 h, 8 h,12 h and *ex vivo* brain imaging at 12 h from ICH mice administrated with free DiR, DiR@PEOz, DiR@NPEOz. (**B**) Radiant efficiency of fluorescence intensity *in vivo* at 1 h, 4 h, 8 h, 12 h from ICH mice administrated with free DiR, DiR@PEOz, DiR@NPEOz (n = 3). (**C**) Radiant efficiency of fluorescence intensity of brain *ex vivo* at 12 h from ICH mice administrated with free DiR, DiR@PEOz, DiR@NPEOz (n = 3). All data are presented as means ± SD. * versus indicated groups, *** *P* < 0.001.

The blood-brain barrier (BBB) is a physiologic barrier that not only maintains intracerebral homeostasis, but also restricts the access of most drugs to the brain parenchyma, which impedes the therapy of brain disorders to a great extent 35, 36. Although the integrity of the BBB was partially compromised after ICH, which offered a probability for therapeutic agents entering the lesion situs, the accumulation of drugs in the brain relying on passively temporary openness of the BBB only slightly increased due to the timing and degree of the BBB opening varied highly with the severity of hemorrhage 10, 72, 73. Inflammatory cells could actively be recruited to the lesions through chemotactic receptors on their membrane surface which interacted with corresponding ligands overexpressed on stressed endothelial cells at the damaged site. As the most principal inflammatory cells, neutrophils rapidly infiltrated into the brain lesions in an initiative manner. Camouflaging with neutrophil membranes has been reported to endow nanoplatforms with the ability of accurately targeting inflammatory lesions 61. To confirm whether neutrophile membranes camouflaging enhanced the nanocarriers' ability of targeting the hemorrhagic brain sites, different forms of DiR including the free DiR, DiR@PEOz and DiR@NPEOz were intravenously injected into ICH mice to trace their fate with help of *In Vivo* Imaging System (IVIS). As illustrated in **Figure 5A**-**B**, the fluorescence signal accumulated prominently as early as at 4 h after treatment with the DiR@NPEOz and remained at a high level for the following time. This observation aligned with the fact that neutrophils would be recruited and infiltrate into the inflammatory hemorrhagic brains within hours 71. In addition, we isolated the brain and main organs at 12 h post-administration to observe the nanoparticles distribution. While a plenty of nanoparticles were taken up by the liver, considerable amounts were still observed in brain tissues (**Figure 5A and S8A**-**B**). DiR@NPEOz elicited a stronger efficiency in targeting delivery to the injured brain, as evidenced by nearly five times higher radiant efficiency in quantitative analysis in DiR@NPEOz group compared to the free DiR or DiR@PEOz group (**Figure 5C**). Taken together, the above data effectively supported that neutrophil membrane camouflaging facilitated BBB penetration of nanoplatforms to inflammatory brain sites where the loaded drugs worked.

### Pro-efferocytic D&G@NPEOz promote hematoma resolution and neurobehavioral recovery after ICH

**Figure 6:**
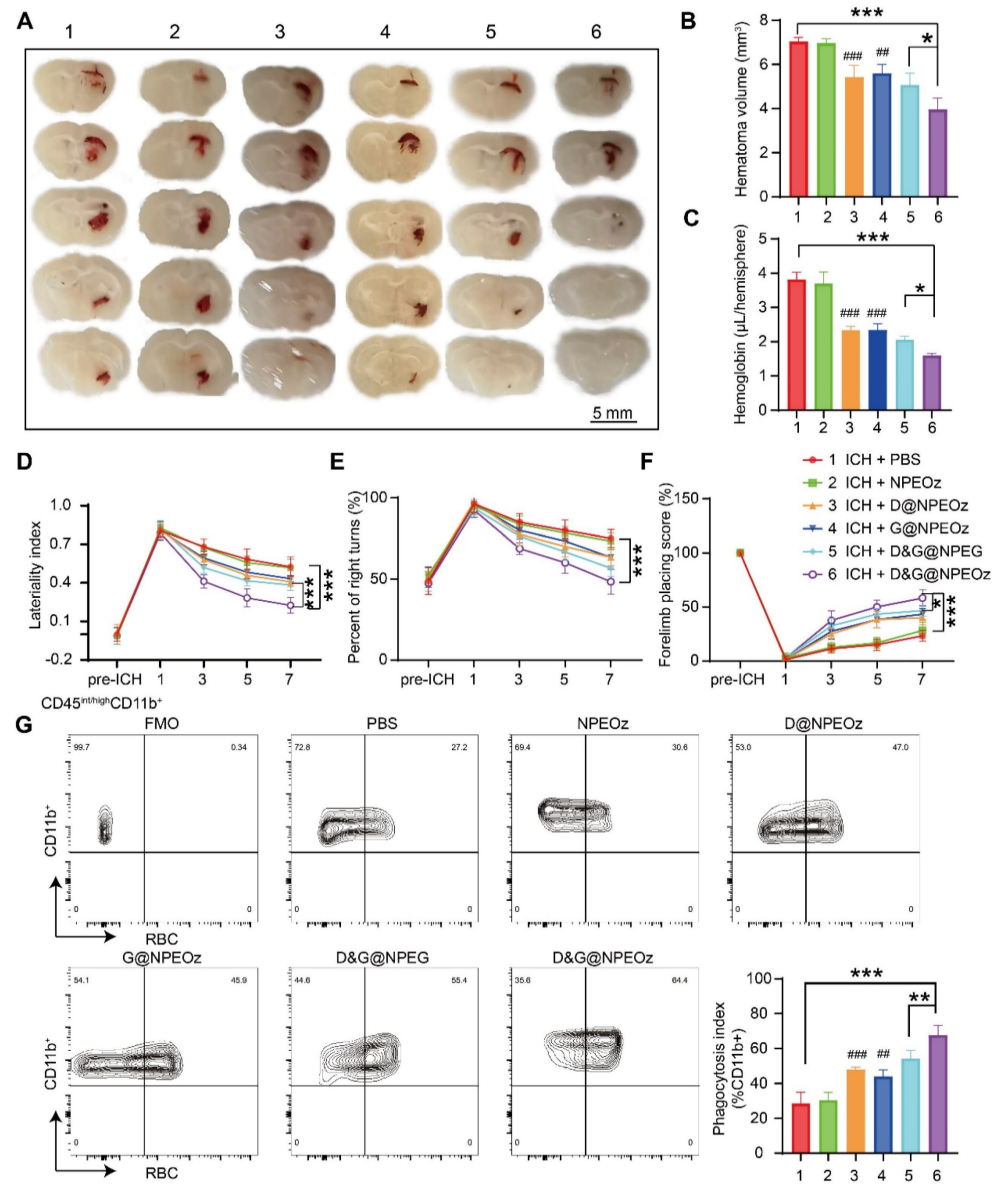
**Pro-efferocytic D&G@NPEOz promote hematoma resolution and neurobehavioral recovery after ICH.** (**A**) Representative brain coronal sections showing the residual hematoma from the mice treated with different formulations at the day 3 after ICH. (**B-C**) Quantification of residual hematoma volume and hemoglobin at day 3 after ICH (n = 4). (**D-F**) Behavioral test results of cylinder test, corner turn and forelimb placement test from the mice treated with different formulations at different time (n = 6-8). (**G**) Representative flow cytometry plots and quantitation analysis (the bottom of right corner) of erythrophagocytosis as CD45^int/high^CD11b^+^ containing labeled-RBCs (n = 4). All data are presented as means ± SD. # versus ICH + PBS group, ##*P* < 0.01, ### *P* < 0.001; * versus indicated groups, *P < 0.05, **P < 0.01, *** *P* < 0.001.

Autologous blood injection is a highly reproducible and reliable animal model due to controllable blood volume, although it does not perfectly reflect the heterogeneity and complexity of ICH patients 74, 75. Allowing the production of homogenous hematomas volumes makes it appropriate to compare the size of the hematoma. Therefore, an intracerebral hemorrhage model induced by autologous injection of blood was chosen to evaluate the effect of NPs on the hematoma resolution. According to the previously published data 4, 8, we chose to value the effect of D&G@NPEOz on erythrophagocytosis at day 3 after ICH. ICH mice were randomly assigned to different treatment groups including PBS, NPEOz, D@NPEOz, G@NPEOz, D&G@NPEG and D&G@NPEOz. The residual hematoma volume was calculated to be severally 7.04 ± 0.19, 6.96 ± 0.20, 5.43 ± 0.53, 5.60 ± 0.41, 5.08 ± 0.52, 3.96 ± 0.52mm^3^ in the PBS, NPEOz, D@NPEOz, G@NPEOz, D&G@NPEG and D&G@NPEOz group. A 27.2% or 29.3% decrease in the volume of hematoma was realized for D&G@NPEOz treatment relative to D@NPEOz or G@NPEOz treatment, respectively (**Figure 6A**-**B**). Accordingly, the hemoglobin levels from mice in D@NPEOz, G@NPEOz, D&G@NPEG and D&G@NPEOz groups dramatically decreased compared with the PBS group. The best efficacy was also obtained by D&G@NPEOz as evidence that D&G@NPEOz-treated mice were observed to have the least levels of hemoglobin among all groups (**Figure 6C**). These data indicated the superior promotion effect of D&G@NPEOz on hematoma absorption. Accelerating erythrophagocytosis prior to erythrocyte lysis and subsequently reducing the consequent release of the detrimental erythrocyte metabolites is beneficial to improve neurological function after ICH. We then performed a series of behavioral tests (the cylinder, corner turn and forelimb placement tests) respectively at day 1,3,5,7 to trace neurological recovery after ICH onset. The degree of deviation from the base point reflected the severity of neurological impairment. As demonstrated in **Figure 6D-F**, there were no differences in the behavioral tests at pre-ICH time, significant deviation from base point occurred at day 1 after ICH onset. From day 3 on, the values of laterality index, percentage of right turns and forelimb placing score from mice were gradually close to the base point in D@NPEOz, G@NPEOz, D&G@NPEG and D&G@NPEOz groups with varied degree, indicating neurological function of mice were improved in these groups. D&G@NPEOz-treatment mice achieved the most potent performance in ameliorating the neurological function as the demonstration that the values of the three tests were the closest to the base point at the study endpoint. As the professional phagocytes with a common lineage, microglia and macrophages, were recruited to clear damaged cells including erythrocytes in the hematoma where a large number of eryptosis had been observed at day 3 after ICH 2. Next, we conducted erythrophagocytosis measurement* in vivo* after ICH through injecting fluorescently labeled RBCs. Three days after ICH, the single-cell suspensions from mice striatal were collected. And then microglia/macrophages (defined as CD45^int/hi^CD11b^+^) were identified by flow cytometry (**Figure S9**). Quantitative analysis results of flow cytometry indicated the percentages of RBCs-engulfing microglia/macrophages (LIVE/DEAD^-^CD45^int/hi^CD11b^+^RBC^+^) in D@NPEOz, G@NPEOz, D&G@NPEG and D&G@NPEOz groups were 47.9%, 44.1%, 54.3%, 67.6% respectively, which were higher than 28.5% observed in the PBS group (**Figure 6G**). Notably, mice treated with D&G@NPEOz exhibited a markedly higher percentage of RBC-positive microglia/macrophages compared to those treated with the no-responsive nano-formulation or single drug-loaded nanoparticles. Collectively, these data demonstrated the co-delivery of GW280264X and desmosterol and pH-triggered drug release both contributed to the heightened therapeutic potency of our nano-preparation.

**Figure 7:**
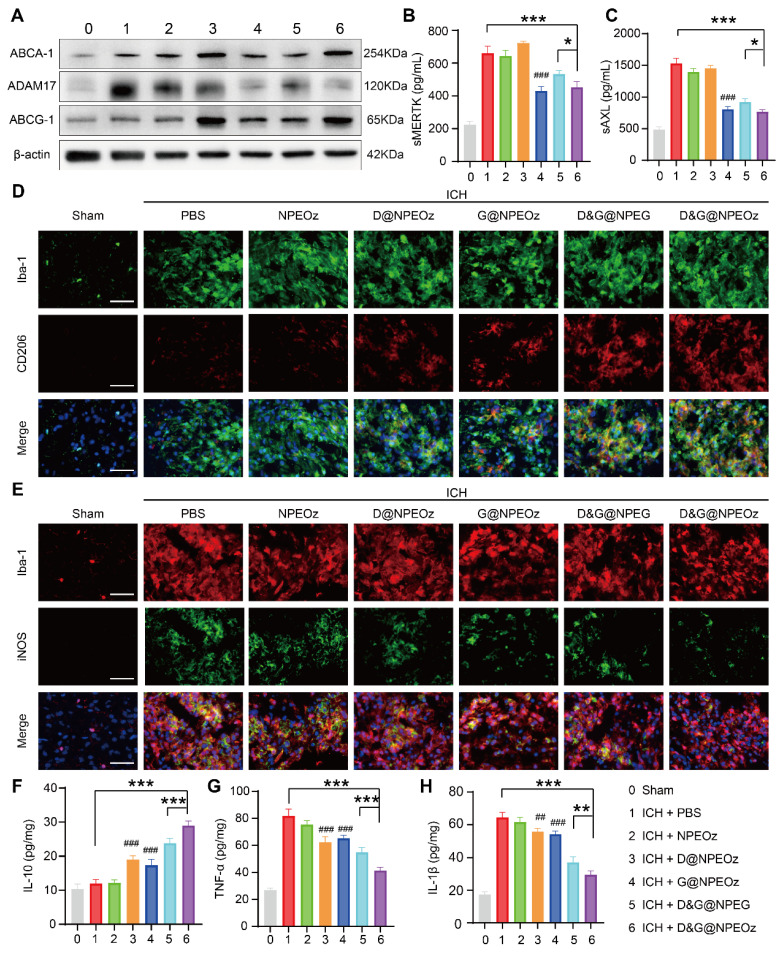
** The mechanism of Pro-efferocytic action and the regulation of proinflammatory microenvironment by D&G@NPEOz NPs *in vivo*.** (**A**) Western blot images showing the expression levels of ABCA-1, ADAM17, ABCG-1 in the peri-hematoma brain tissues from the mice treated with different formulations at the day 3 post ICH. (**B-C**) The levels of sMERTK and sAXL in serum from the mice treated with different formulations at day 3 post-ICH (n = 4). (**D**) Representative immunofluorescence staining with Iba-1 (green, a microglia marker), CD206 (red, a M2 marker) in the ipsilateral striatum around the hematoma from the mice treated with different formulations (Scale bar = 50 μm). (**E**) Representative immunofluorescence staining with Iba-1 (red), iNOS (green, a M1 marker) in the ipsilateral striatum around the hematoma from the mice treated with different formulations (Scale bar = 50 μm). (**F-H**) The levels of IL-10, TNF-α and IL-β in the peri-hematoma brain tissues from the mice treated with different formulations at day 3 post-ICH (n = 4). All data are presented as means ± SD. # versus ICH + PBS group, ##*P* < 0.01, ### *P* < 0.001; * versus indicated groups, *P < 0.05, **P < 0.01, *** *P* < 0.001.

To further validate the specific molecular mechanisms of D&G@NPEOz treatment *in vivo*, the activity and protein expression of ADAM17 from brain tissues within the peri-hematoma region as well as the level of sMERTK and sAXL in the plasma were firstly investigated. Similar to the results *in vitro*, both G@NPEOz and D&G@NPEOz treatments led to a significant decrease in the protein expression and enzymatic activity of ADAM17 in brain tissues compared to PBS treatment. (**Figure 7A** and** S10**). Additionally, the level of sMERTK and sAXL of the plasma were also dramatically declined (**Figure 7B**-**C**). In the meanwhile, we examined the protein expression of ABCA1 and ABCG1. It was noted that the level of ABCA1 and ABCG1 increased in mice from desmosterol-loaded paraparticles D@NPEOz and D&G@NPEOz treatment groups in comparison to the PBS group (**Figure 7A**). Consistent with our earlier findings *in vitro*, these results indicated that the synergy of ADAM17 inhibition combined with LXR activation acted as an impressive booster for erythrocyte clearance after ICH.

### Pro-efferocytic D&G@NPEOz alleviates inflammation *in vivo*

Given that D&G@NPEOz modulated the pro-inflammatory environment to the anti-inflammatory environment, including promoting the formation of reparative M2 phenotype and triggering changes of inflammation-related factors cytokines *in vitro*. We were interested in validating whether D&G@NPEOz exerted similar beneficial effects *in vivo*. Immunostaining assays of brain sections were histologically applied to reveal the phenotypic transformation of microglia/macrophages after treatment with different nano-preparations. The results of immunofluorescence staining illustrated that Iba-1 was highly expressed in the ipsilateral striatum around the hematoma, indicating microglia/macrophage proliferation after ICH. The M1 and M2 subtypes of microglia/macrophage were confirmed as iNOS^+^/Iba-1^+^and CD206^+^/Iba-1^+^, respectively. It was observed that the M2 phenotype marker CD206 increased (**Figure 7D**) while the M1 phenotype marker iNOS (**Figure 7E**) decreased in all drug-loaded nanoparticle treatment groups. Moreover, there were much more CD206^+^/Iba-1^+^cells and fewer iNOS^+^/Iba-1^+^ cells found in the D&G@NPEOz treatment group in comparison with other nanoparticle treatment groups. We next measured the levels of proinflammatory and anti-inflammatory cytokines in brain tissue from the peri-hematoma areas by ELISA. In line with the results *in vitro*, we observed that compared with the PBS-treated group, D&G@NPEOz treatment increased the expression of the anti-inflammatory cytokines IL-10 by 140% (**Figure 7F**), decreased secretion of the proinflammatory cytokines TNF-α by 49.4% and IL-β by 54.1%, respectively (**Figure 7G**-**H**), with the most notable effect among all groups tested. In summary, our study highlighted the potential therapeutic role of D&G@NPEOz in mitigating brain injury induced by ICH through its ability to facilitate microglia/macrophage polarization from an M1 to an M2 phenotype. This modulation led to a decrease in proinflammatory cytokine production and an increase in anti-inflammatory cytokine secretion, ultimately promoting inflammation resolution. Therefore, D&G@NPEOz holds promise as a valuable strategy for managing ICH-induced inflammatory microenvironment.

### *In vivo* safety evaluation

**Figure 8:**
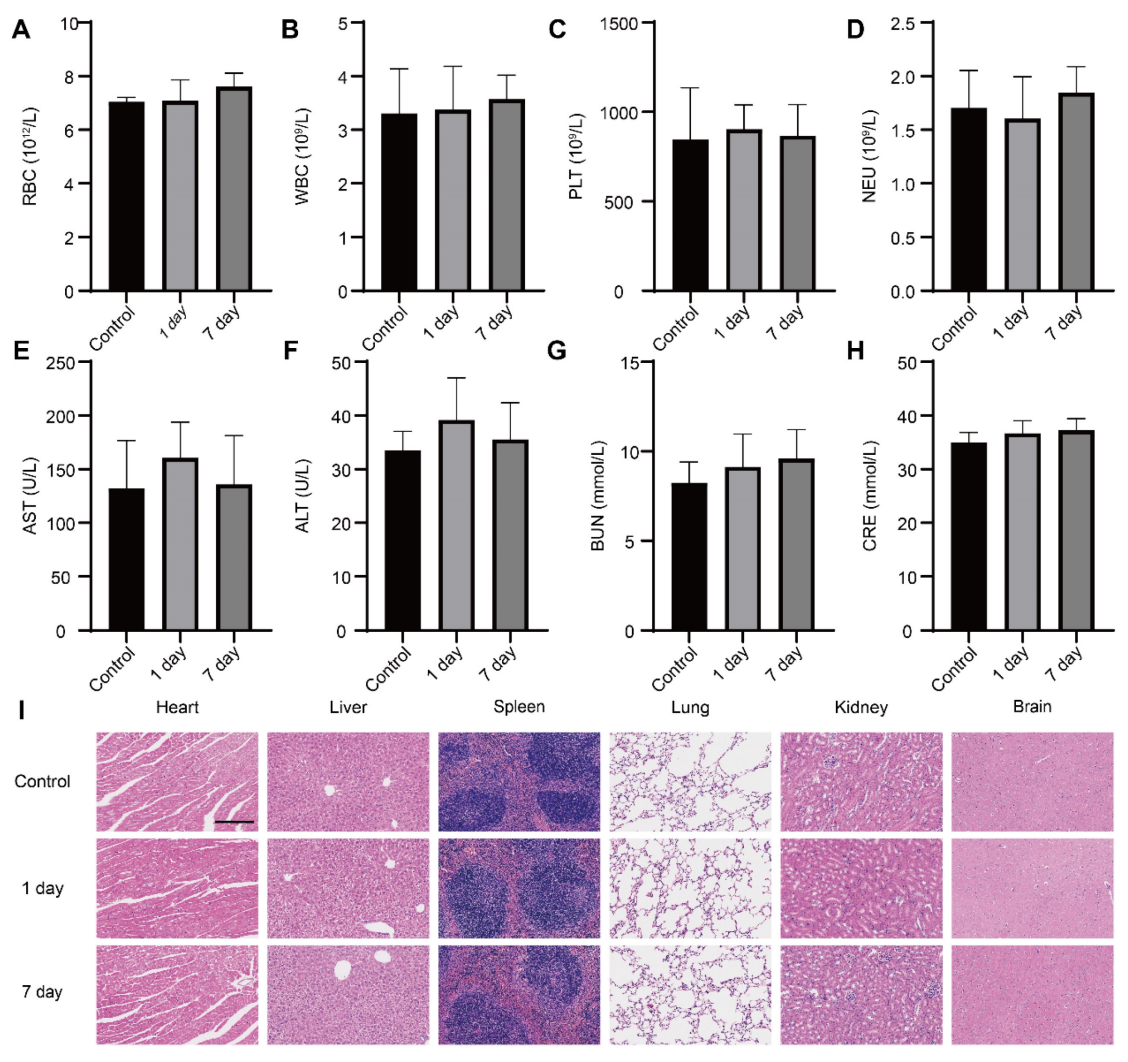
** Safety evaluation *in vivo*.** (**A-D**) The levels of routine blood indexes (red blood cell, white blood cells, platelets and neutrophil) from mice at day 1 and day 7 post-injection D&G@NPEOz nanoparticles (n = 4). (**E-H**) The levels of common blood biochemical parameter (alanine aminotransferase, aspartate aminotransferase, blood urea nitrogen and creatinine) from mice at day 1 and day 7 post-injection D&G@NPEOz nanoparticles (n = 4). (**I**) Representative H&E staining images of main organs (heart, liver, spleen, lung, kidney, and brain) on day 1 and day 7 post-injection D&G@NPEOz nanoparticles (Scar bar = 200 μm). All data are presented as means ± SD.

We conducted a comprehensive investigation into the systemic toxicity of nanoparticles. In comparison to control mice, routine blood indexes including RBC, WBC, PLT and Neu were within the normal range at both day 1 and day 7 post-injection (**Figure 8A-D**), indicating good compatibility of the nanoparticles. The negligible toxicity of the nanoparticles was further conformed by the absence of obvious deviations in the common blood biochemical parameters such as AST, ALT, GRE and BUN (**Figure 8E-H**), which suggested the nanomedicine did not impair the functions of the mice liver and kidney. Furthermore, there were no distinguishable abnormalities noticed in hematoxylin-eosin (H&E) staining images from the brain, heart, liver, spleen, lung and kidney (**Figure 8I**), hinting intravenous administration of nanoparticles did not result in striking toxicity in major organs. The above results provided support for considering the as-prepared nanoparticle as a safe candidate for therapy of brain disease.

## Summary and discussion

The cascaded damage mechanisms after ICH provide a clue that how to alleviate the injury of the brain resulting from blood extravagating into the brain parenchyma should be the focus due to the fact that the downstream mechanisms of ICH damage are all rooted from the extravasated blood. More attention is deservedly attracted to the removal of cerebral hematoma, which is the most promising strategy for the management of ICH. However, most of the existing studies concentrate on the downstream events after ICH 10, 76-78. There were few researches about endogenous clearance of hematoma, which mainly focused on how to enhance interaction between microglia/macrophage and erythrocytes by regulating the associated signaling pathways, such as promoting the expression of phagocytic receptors or reducing phagocytosis-inhibiting signal 4, 7, 79-81. The process by which phagocytes deal with phagocytic cargoes was often overlooked. Indeed, as a type of efferocytosis, phagocytic clearance of erythrocytes after ICH is a complicated process that is not just simplistic communication between efferocytes and erythrocytes. For efficient erythrophagocytosis, a single phagocyte has to take up multiple apoptotic erythrocytes in view of the fact that erythrocytes much outnumber phagocytes in the hemorrhagic environment 82. It is of great importance that efferocytes effectively process the residues from ingested corpses to avoid the accumulation of large amounts of metabolic cargoes in the process of efferocytosis and make room for the sequent phagocytosis. We anticipated that the combination of maintaining the availability of phagocytic receptors with preventing massive accumulation of cholesterol potentiated erythrophagocytosis to improve recovery after ICH. As we expected, the combined effect of ADAM17 inhibition and LXR activation was obviously stronger than that of any single one, indicating their synergy acted as an impressive booster for erythrocyte clearance and neurological recovery after ICH.

How to increase the accumulation of therapeutic drugs at desired sites is another key issue in achieving an encouraging effect. On the one hand, many small-molecule drugs possess poor bioavailability due to low solubility and off-target effect. On the other hand, BBB restricts almost all of large-molecule therapeutic drugs and more than 98% of small-molecule agents access to the brain 83. Neutrophil membrane camouflaging has been reported to be a promising solution to accurately deliver nanoparticles to insult lesions by the interaction between their surface chemotactic receptors and the corresponding ligands overexpressed on VECs 59, 60, 63. Consistent with the published results, our synthetic nanoparticles inherited the key chemotactic proteins from neutrophils and efficiently accumulated at the hemorrhagic sites after intravenous injection, which offered an excellent opportunity for improving the efficiency of our hydrophobic drugs with diminished side effects in the management of ICH.

## Conclusion

In conclusion, we successfully developed neutrophil-disguised pH-responsive nanoparticles to efficiently co-deliver GW280264X and desmosterol to accelerate hematoma removal by synergistically promoting erythrophagocytosis. The biomimetic nanocarriers elicited favorable biocompatibility and the ability of targeting injury sites inherited from the neutrophil. The nanoparticles could accurately accumulate at hemorrhagic sites and responsively release therapeutic GW280264X and desmosterol under acidic environments. The combination of GW280264X and desmosterol enhanced erythrophagocytosis through inhibiting shedding of the efferocytotic receptors MERTK/AXL mediated by ADAM17 and accelerating ABCA-1/ABCG-1-mediated cholesterol efflux regulated by LXR respectively. In addition, the nano-formulation was able to modulate the inflammatory microenvironment by promoting the restorative phenotypic transition of microglia/macrophage, increasing the levels of the anti-inflammatory cytokine IL-10, decreasing the expression of pro-inflammatory factors TNF-α and IL-1β, and improve neurological function. This biomimetic nanomedicine is envisaged to offer an encouraging strategy to effectively promote hematoma and inflammation resolution, consequently alleviate ICH progression.

## Supplementary Material

Supplementary figures.
