# Supplementary material for: Neutrophil-like pH-responsive pro-efferocytic nanoparticles improve neurological recovery by promoting erythrophagocytosis after intracerebral hemorrhage
Source: Theranostics. 2024 Jan 1;14(1):283–303. doi: 10.7150/thno.90370 (PMC10750197; doi:10.7150/thno.90370)
Supplement: Supplementary file 1 — Supplementary figures. [file thnov14p0283s1.pdf]

Supporting information

Neutrophil-like pH-responsive pro-efferocytic nanoparticles improve neurological recovery by promoting erythrophagocytosis after intracerebral hemorrhage

Author names: Linfeng Fan[1,2,#], Lulu Jin[3,#], Tianchi Tang[1,2,#], Yonghe Zheng[1,2], Zihang Chen[1,2], Haopu Lin[1,2], Chao Ding[1,2], Tingting Wang[1,2], Huaijun Chen[1,2], Yinghan Guo[1,2], Chaoran Xu[1,2], Hang Zhou[1,2], Xinyan Wu[1,2], Xiongjie Fu[1,2], Feng Yan[1,2,*], Zhengwei Mao[3,*], Gao Chen[1,2,*]

Affiliations: *1. Department of Neurosurgery, The Second Affiliated Hospital of Zhejiang University School of Medicine, Zhejiang University, Hangzhou, 310016, China.*

2. Key Laboratory of Precise Treatment and Clinical Translational Research of Neurological Diseases, Hangzhou, 310016, China.

3. MOE Key Laboratory of Macromolecular Synthesis and Functionalization, Department of Polymer Science and Engineering, Zhejiang University, Hangzhou, 310027, China.

# Linfeng Fan, Lulu Jin and Tianchi Tang *contributed equally to this work.*

***Corresponding author:**

Gao Chen, Department of Neurosurgery, The Second Affiliated Hospital of Zhejiang University School of Medicine, No.88 Jiefang Road, Hangzhou, China. Email address: d-chengao@zju.edu.cn

Zhengwei Mao, MOE Key Laboratory of Macromolecular Synthesis and Functionalization, Department of Polymer Science and Engineering, Zhejiang University, Hangzhou, 310027, China Email address: zwmao@zju.edu.cn

Feng Yan, Department of Neurosurgery, The Second Affiliated Hospital of Zhejiang University School of Medicine, No.88 Jiefang Road, Hangzhou, China. Email address: fengyanzju@zju.edu.cn.

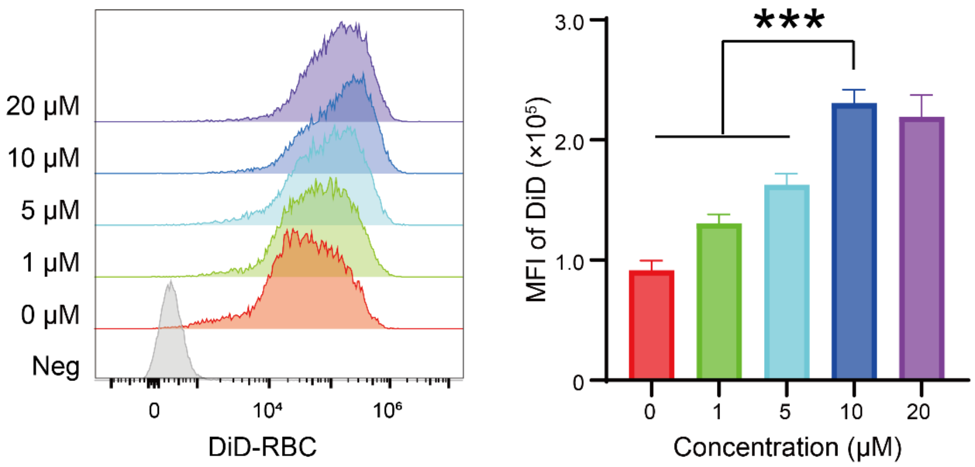

Figure S1. Flow cytometry histogram and quantitative analysis of MFI of engulfed fluorescent-labeled erythrocytes in BV2 cells with different concentrations of desmosterol treatment followed by 2 h incubation with DiD-labeled RBCs (n = 3). All data are presented as means ± SD. * versus indicated groups, *** $P < 0.001$.

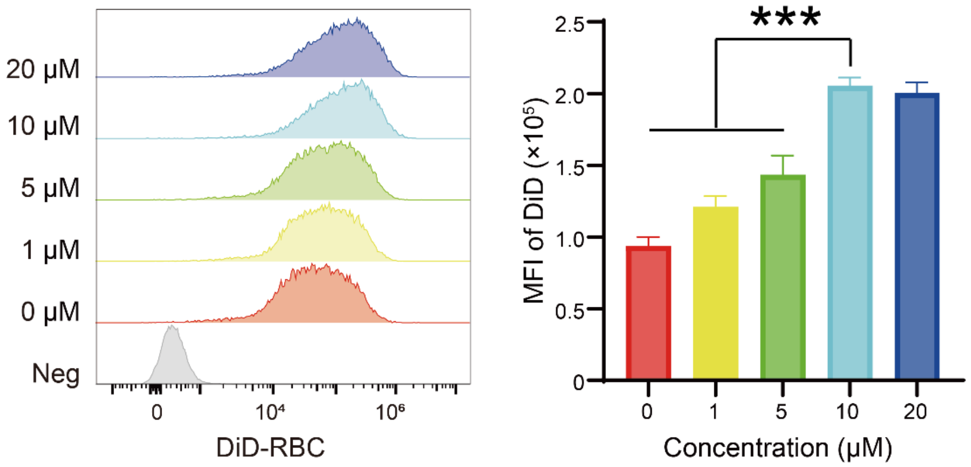

Figure S2. Flow cytometry histogram and quantitative analysis of MFI of engulfed fluorescent-labeled erythrocytes in BV2 cells with different concentrations of GW280264X treatment followed by 2 h incubation with DiD-labeled RBCs (n = 3). All data are presented as means ± SD. * versus indicated groups, *** $P < 0.001$.

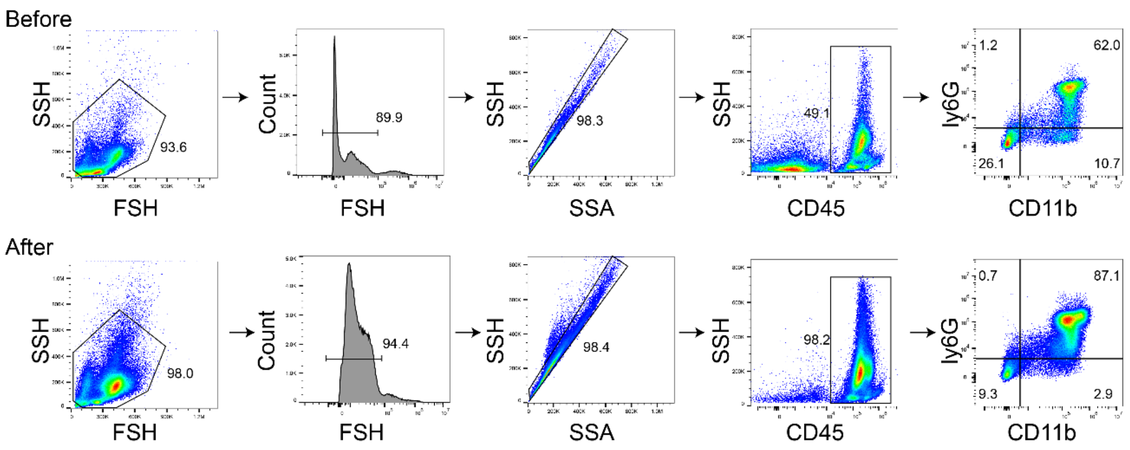

Figure S3. Flow cytometric analysis of the ratios of neutrophils in the cells isolated from bone marrow of C57BL/6 mice through Histopaque 1119/1077 gradient centrifugation.

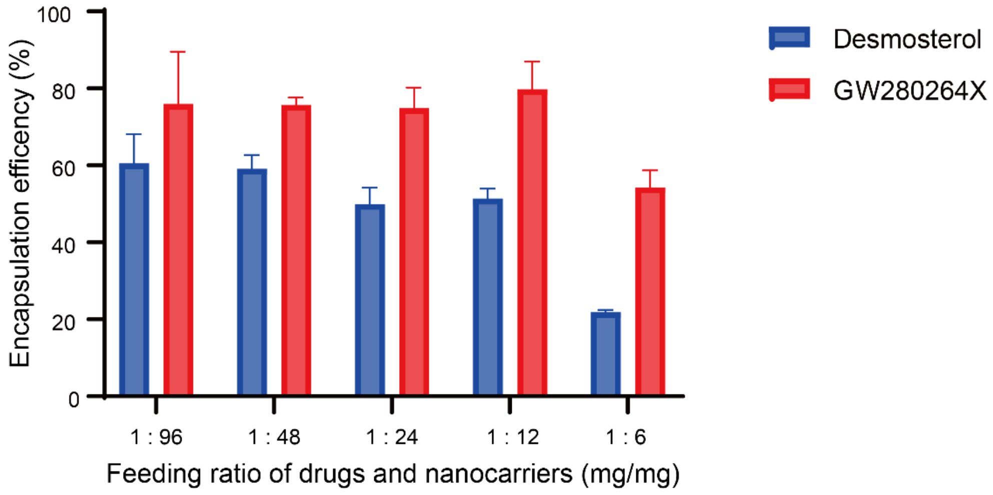

Figure S4. Encapsulation efficiency of D&G@NPEOz at different ratios of drugs and nanocarriers. The molar ratio of desmosterol/GW280264X was 1:1. All data are presented as means ± SD.

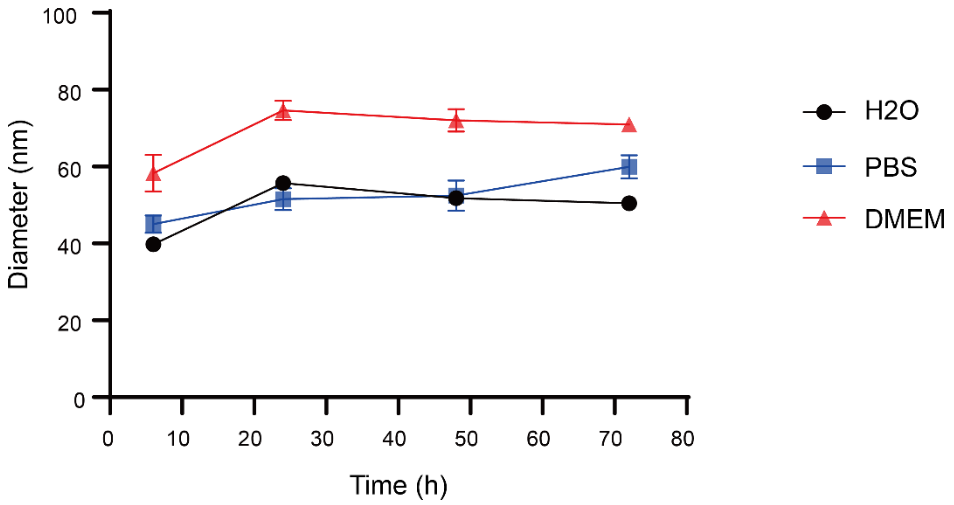

Figure S5. The stability of D&G@NPEOz in H_2O, PBS and DMEM respectively.

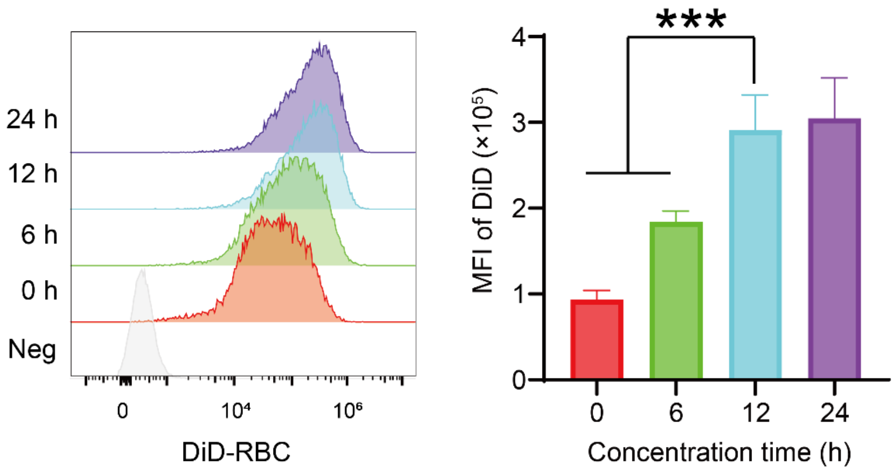

Figure S6. Flow cytometry histogram and quantitative analysis of MFI of engulfed fluorescent-labeled erythrocytes in BV2 cells after different treatment time with D&G@NPEOz followed by 2 h incubation with DiD-labeled RBCs (n = 3). All data are presented as means ± SD. * versus indicated groups, *** $P < 0.001$.

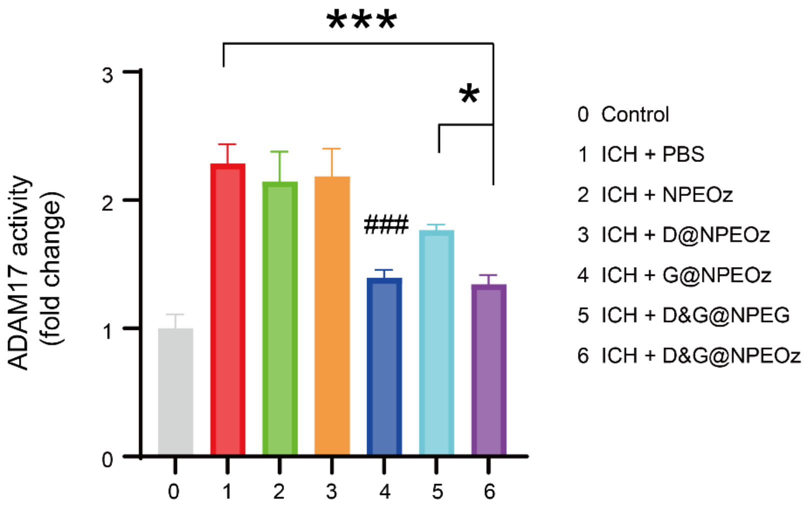

Figure S7. Quantitative analysis of ADAM17 activity in BV2 cells with different formulations treatment (n = 3). All data are presented as means ± SD. # versus ICH+ PBS group, ### $P < 0.001$; * versus indicated groups, *$P < 0.05$, *** $P < 0.001$.

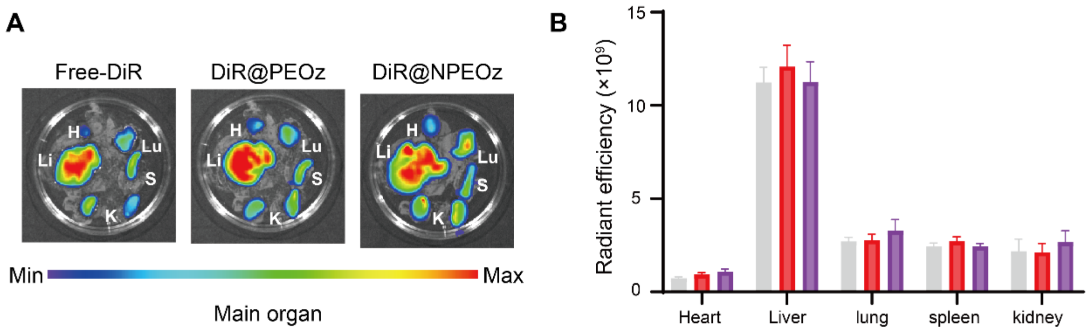

Figure S8. (A) *Ex vivo* images of main organs (H for heart, Li for liver, Lu for lung, S for spleen, K for kidney) at 12 h from ICH mice administrated with free DiR, DiR@PEOz, DiR@NPEOz. (**B**) Radiant efficiency of fluorescence intensity of major organs *ex vivo* at 12 h from ICH mice administrated with free DiR, DiR@PEOz, DiR@NPEOz (n = 3). All data are presented as means ± SD.

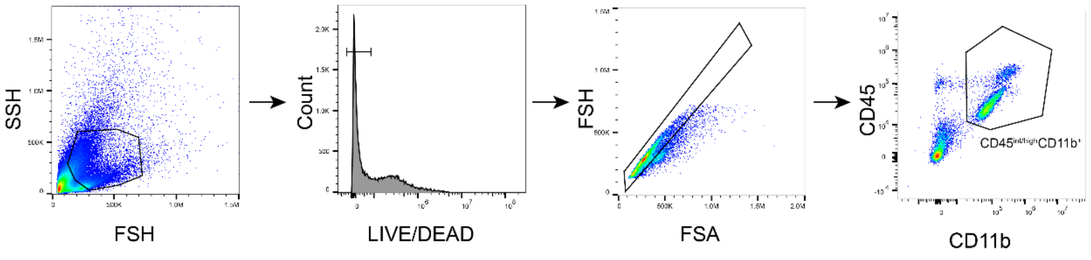

Figure S9. Gating strategy for analysis of erythrophagocytosis in vivo by flow cytometry.

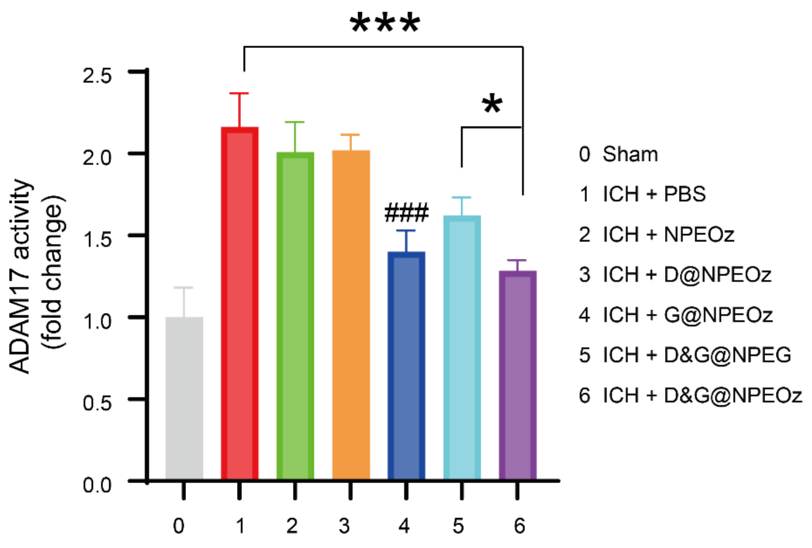

Figure S10. Quantitative analysis of ADAM17 activity in the hematoma region from the mice treated with different formulations at the day 3 post ICH (n = 4). All data are presented as means ± SD. # versus ICH+ PBS group, ### *P* < 0.001; * versus indicated groups, **P* < 0.05, *** *P* < 0.001.
